# The LKB1-salt-inducible kinase pathway functions as a key gluconeogenic suppressor in the liver

**DOI:** 10.1038/ncomms5535

**Published:** 2014-08-04

**Authors:** Kashyap Patel, Marc Foretz, Allison Marion, David G. Campbell, Robert Gourlay, Nadia Boudaba, Emilie Tournier, Paul Titchenell, Mark Peggie, Maria Deak, Min Wan, Klaus H. Kaestner, Olga Göransson, Benoit Viollet, Nathanael S. Gray, Morris J. Birnbaum, Calum Sutherland, Kei Sakamoto

**Affiliations:** 1 MRC Protein Phosphorylation and Ubiquitylation Unit, College of Life Sciences, University of Dundee, Dow Street, Dundee DD1 5EH, UK; 2 INSERM, U1016, Institut Cochin, 75014 Paris, France; 3 CNRS, UMR8104, 75014 Paris, France; 4 Université Paris Descartes, Sorbonne Paris Cité, 75006 Paris, France; 5 The Institute for Diabetes, Obesity, and Metabolism, University of Pennsylvania, Philadelphia, Pennsylvania 19104, USA; 6 The Department of Experimental Medical Science, Lund University, BMC C11, 221 84 Lund, Sweden; 7 Department of Biological Chemistry and Molecular Pharmacology, Harvard Medical School, Dana–Farber Cancer Institute, Boston, Massachusetts 02115, USA; 8 Cardiovascular and Diabetes Medicine, Ninewells Hospital and Medical School, University of Dundee, Dundee DD1 9SY, UK; 9 These authors contributed equally to this work; 10 Present address: Nestlé Institute of Health Sciences SA, EPFL Innovation Park, bâtiment G, 1015 Lausanne, Switzerland

## Abstract

LKB1 is a master kinase that regulates metabolism and growth through adenosine monophosphate-activated protein kinase (AMPK) and 12 other closely related kinases. Liver-specific ablation of LKB1 causes increased glucose production in hepatocytes *in vitro* and hyperglycaemia in fasting mice *in vivo*. Here we report that the salt-inducible kinases (SIK1, 2 and 3), members of the AMPK-related kinase family, play a key role as gluconeogenic suppressors downstream of LKB1 in the liver. The selective SIK inhibitor HG-9-91-01 promotes dephosphorylation of transcriptional co-activators CRTC2/3 resulting in enhanced gluconeogenic gene expression and glucose production in hepatocytes, an effect that is abolished when an HG-9-91-01-insensitive mutant SIK is introduced or LKB1 is ablated. Although SIK2 was proposed as a key regulator of insulin-mediated suppression of gluconeogenesis, we provide genetic evidence that liver-specific ablation of SIK2 alone has no effect on gluconeogenesis and insulin does not modulate SIK2 phosphorylation or activity. Collectively, we demonstrate that the LKB1–SIK pathway functions as a key gluconeogenic gatekeeper in the liver.

## Abstract (web summary)

The liver is an important regulator of glucose homeostasis. Here, the authors provide insight into the molecular signalling pathways controlling hepatic gluconeogenesis by showing that SIK protein kinases suppress gluconeogenesis, and that glucagon—but not insulin—regulates phosphorylation of SIK2.

## Graphical abstract

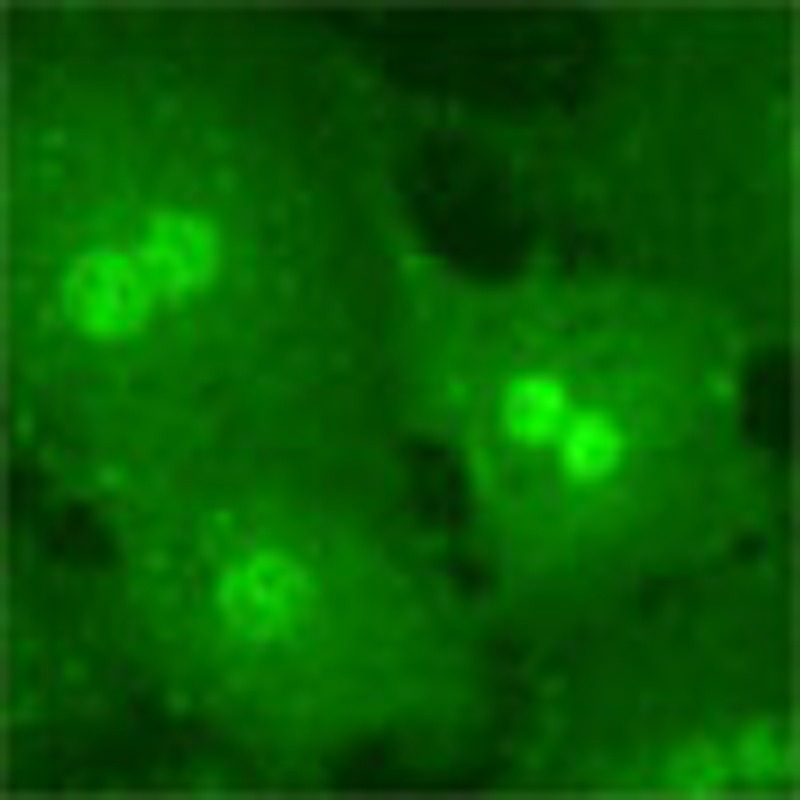

Blood glucose concentration is tightly controlled in healthy humans to prevent the toxic effects of prolonged hyperglycaemia (diabetic complications) and the acute debilitating effects of hypoglycemia (coma). Two vital components to this control are the suppression of hepatic glucose output following a meal, and the induction of hepatic glucose production during prolonged fasting. The latter involves increased breakdown of stored hepatic glycogen and *de novo* synthesis of glucose from substrates including glycerol, lactate and amino acids (gluconeogenesis), both in response to glucagon released from pancreatic α-cells when blood glucose falls. In the postprandial state, these processes are largely suppressed by insulin released from pancreatic β-cells and the concomitant decrease in glucagon in response to elevated blood glucose and glucose itself. Deregulation of one or more of these processes contributes to development of metabolic disorders such as type 2 diabetes. Therefore delineating the molecular control of glucose homoeostasis is fundamental to combating the rising tide of metabolic disease in modern populations.

A key point of control of hepatic gluconeogenesis is transcriptional regulation of genes encoding rate-controlling enzymes, including the catalytic subunit of glucose-6-phosphatase (G6Pase, encoded by the *G6pc* gene) and cytosolic phosphoenolpyruvate carboxykinase (PEPCK, encoded by the *Pck1* gene). Two major signalling pathways that suppress gluconeogenic gene transcription are the insulin signalling pathway and also putatively the LKB1/adenosine monophosphate-activated protein kinase (AMPK) pathway. Substantial evidence supports a model in which insulin, via Akt-mediated phosphorylation and inhibition of forkhead box protein-O (FoxO), reduces the expression of *G6pc* and *Pck1* (ref. 1). AMPK is a key intracellular energy sensor and regulator of multiple metabolic processes^2^. Zhou *et al.*^3^ demonstrated that metformin (the most widely prescribed drug for glycaemic control in people with type 2 diabetes) stimulated AMPK and this was associated with inhibition of glucose production in rat primary hepatocytes. Therefore AMPK was proposed as a potential regulator of hepatic gluconeogenesis. More recently we tested directly whether the effect of metformin on hepatic gluconeogenesis required AMPK by investigating metformin action in a mouse model lacking both AMPKα1 and α2 catalytic subunits in hepatocytes^4^. Importantly liver-specific AMPKα1/α2-null mice were normoglycaemic in both fasted and fed states, and an acute administration of metformin to the AMPKα1/α2-knockout (KO) mice resulted in glucose lowering equivalent to the wild-type (WT) control animals. Moreover, primary hepatocytes lacking AMPK treated with metformin displayed a robust inhibition of glucose production. In addition, we found that the pharmacological AMPK activator, A-769662 (that directly activates AMPK independently of canonical AMP-binding sites)^5^,^6^,^7^, had no effect on glucose production. However, the less specific AMPK activator 5*-*aminoimidazole*-*4*-*carboxamide*-*1*-*β*-*D*-*ribofuranoside (AICAR), an AMP mimetic (AICA ribonucleotide, ZMP)^8^, profoundly inhibited glucose production even in hepatocytes derived from AMPKα1/α2-null mice^4^. These data implicate AMP *per se* but indicate that AMPK is not required for suppression of hepatic glucose production in response to metformin or feeding. Interestingly, there is now evidence for mitochondrial inhibition (independent of AMPK) influencing the action of the cyclic AMP (cAMP)-dependent protein kinase (also known as protein kinase A (PKA)) signalling pathway in the regulation of hepatic gluconeogenesis^9^,^10^.

Although loss of AMPK does not alter regulation of hepatic gluconeogenesis, ablation of hepatic LKB1, a major upstream kinase of AMPK, was associated with hyperglycaemia in mice^11^. There was also a higher rate of basal/unstimulated and cAMP-stimulated glucose production in LKB1 KO hepatocytes compared with control cells^4^. LKB1 is a master kinase that directly phosphorylates the activation loop of AMPKα1, α2 and another 12 kinases related to AMPK (thus called AMPK-related kinases), including the salt-inducible kinase isoforms (SIK1/2/3; refs 12, 13). As its name suggests, SIK was cloned from adrenal glands of rats fed with a high-salt diet^14^. The activity of all three SIK isoforms is regulated through phosphorylation of a conserved threonine residue in the T-loop of the kinase domain, a phosphorylation which is essential for catalytic activity^12^. Interestingly, it has been reported that SIK2 activity can also be modulated by phosphorylation outside of the T-loop/kinase domain. Indeed these alternative phosphorylation sites were proposed to be critical in controlling the gluconeogenic gene programme (*Pck1* and *G6pc*) by regulating phosphorylation of the cAMP-response element-binding protein (CREB)-regulated transcription co-activator-2 (CRTC2) by SIK2 (ref. 15). Dentin *et al.*^16^ proposed that SIK2 is a key enzyme in the pathways that switch off hepatic gluconeogenesis in response to feeding/insulin. They reported that insulin activated SIK2 through induction of Ser358 phosphorylation downstream of phosphoinositide 3-kinase (PI3K)–Akt signalling. Activated SIK2 in turn stimulated CRTC2 phosphorylation and cytosolic translocation, thereby disrupting CRTC2/CREB-induced gluconeogenic gene transcription. The mechanism by which phosphorylation of Ser358 promotes catalytic activity of SIK2 was not described. In contrast, a recent study has demonstrated that in 3T3-L1 and rat primary adipocytes, agents that increase intracellular cAMP levels promoted Ser358 phosphorylation on SIK2, without any alteration of SIK2 catalytic activity measured *in vitro*^17^. Importantly, in contrast to Dentin *et al.*^16^, they did not observe induction of Ser358 phosphorylation on SIK2 in response to insulin. Therefore the above studies have not reached a consensus on how hormones such as insulin and glucagon regulate SIK2. Clearly, hormones/agonists that stimulate cAMP/PKA and/or PI3K–Akt in cells promote phosphorylation of SIK2 at multiple sites; however, since the discrepancies described above suggest cell/tissue-specific aspects to the regulation of SIK2, it is imperative to study these processes in precise detail in the liver, the key site of abnormal gluconeogenesis in metabolic disease.

In the current study, we demonstrate that potent and selective SIK inhibitors promote dephosphorylation of CRTC2/3, resulting in increased gluconeogenic gene expression and glucose production in mouse primary hepatocytes, which is abolished when the drug/inhibitor-resistant mutant of SIK2 is introduced. Both the SIK inhibitor and glucagon increase glucose production in LKB1 control hepatocytes, but only glucagon has the effect in LKB1 KO hepatocytes. Although SIK2 was proposed as a key regulator for insulin-mediated suppression of gluconeogenesis via phosphorylation/activation, we provide genetic evidence that liver-specific ablation of SIK2 alone has no effect on gluconeogenesis and insulin does not modulate SIK2 phosphorylation/activity. Together these data provide evidence that SIK isoforms play a key role as ‘molecular gate-keepers’ for hepatic glucose production by keeping the gluconeogenic programme repressed, and this can be released by fasting/glucagon signals through a pathway at least partially independent of SIKs.

## Results

### Glucagon but not insulin promotes phosphorylation of SIK2

**Figure 1:**
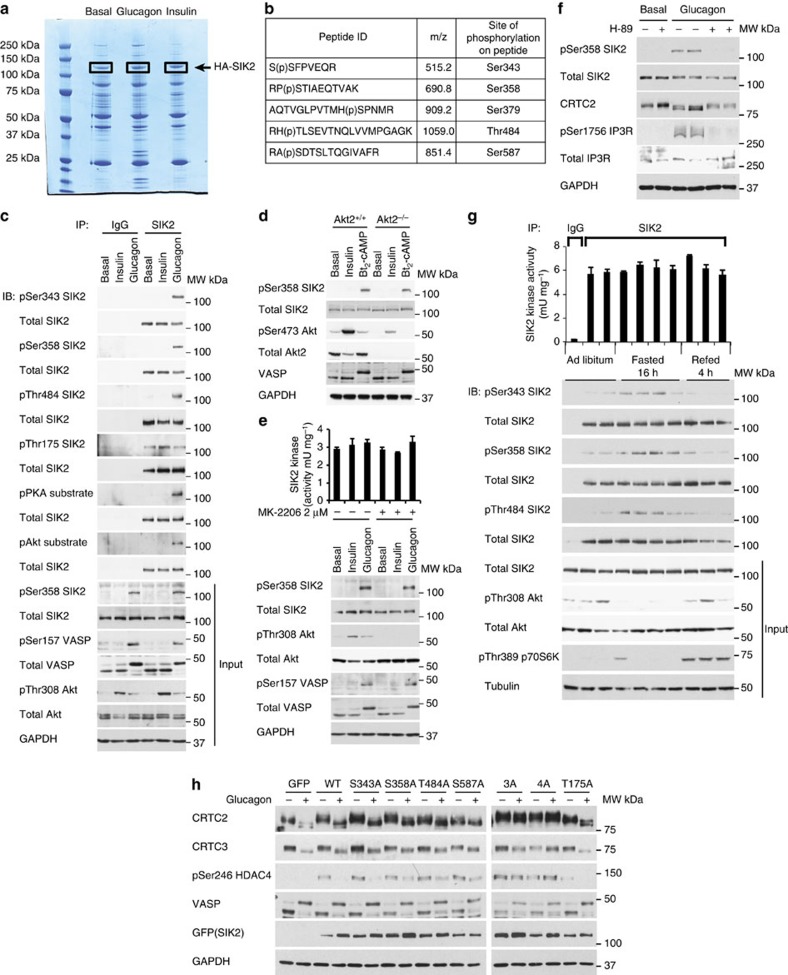
Glucagon but not insulin promotes phosphorylation of SIK2. (**a**) Recombinant HA–SIK2 was expressed in primary mouse hepatocytes (8–12-week-old male C57BL/6) using adenoviral transduction 16 h prior to stimulation with 0.1 μM glucagon or 10 nM insulin for 10 min. HA–SIK2 was immunoprecipitated from 5 mg of lysates with HA-agarose beads and the precipitates were subjected to SDS–polyacrylamide gel electrophoresis followed by colloidal coomassie staining. Bands corresponding to HA–SIK2 (boxed) were excised, in-gel digested with trypsin and analyzed using liquid chromatography mass spectrometry (LC-MS) as described in Methods. (**b**) Table lists the sequences of the phosphopeptides identified, m/z values and respective phosphorylated residues (p) of SIK2 in the glucagon-treated sample. (**c**) Primary hepatocytes were stimulated with either 0.1 μM glucagon or 10 nM insulin for 10 min prior to cell lysis. Endogenous SIK2 was immunoprecipitated from cell extracts and the resultant precipitates were immunoblotted with the indicated antibodies. (**d**) Primary hepatocytes were isolated from Akt2^+/+^ and liver-specific Akt2^−/−^ mice and then stimulated with 10 nM insulin or 100 μM Bt_2_-cAMP for 10 min prior to cell lysis. The lysates were immunoblotted with the indicated antibodies. (**e**) Primary hepatocytes were pre-treated with 2 μM MK-2206 for 1 h followed by 0.1 μM glucagon or 10 nM insulin treatment for 10 min. The lysates were immunoblotted with the indicated antibodies. Endogenous SIK2 was immunoprecipitated and was subjected to *in vitro* kinase assay as described in the Methods. Data is presented as mean±s.d., *n*=3. (**f**) Primary mouse hepatocytes were pre-treated with 25 μM H-89 for 1 h followed by 0.1 μM glucagon treatment for 10 min prior to cell lysis. Lysates were immunoblotted with the indicated antibodies. (**g**) Liver extracts were generated from C57BL/6 male mice (8–10-week-old) that had been subjected to *ad libitum* feeding, 16 h fasting or 16 h fasting followed by 4 h refeeding. Endogenous SIK2 was immunoprecipitated from liver extracts and the precipitates were immunoblotted with the indicated antibodies or subjected to *in vitro* kinase assay. Data is presented as mean±s.d., *n*=3. (**h**) GFP alone, GFP–SIK2 wild-type (WT) or the indicated GFP–SIK2 mutants were expressed in primary hepatocytes using adenoviral vectors (1:2 MOI) 16 h prior to 0.1 μM glucagon treatment for 10 min. The lysates were immunoblotted with the indicated antibodies. The recombinant SIK2 3A protein included S343A, S358A and T484A mutations, while 4A mutant had these mutations plus the S587A mutation.

Recombinant haemagglutinin (HA)-tagged SIK2 was expressed in mouse primary hepatocytes using adenovirus and the cells were left untreated or treated with glucagon or insulin. HA–SIK2 protein was affinity-purified (Fig. 1a), in-gel digested with trypsin and the resulting peptides were analyzed by liquid chromatography-tandem mass spectrometry (LC-MS-MS). We identified several phospho-peptides in a precursor ion scanning LC-MS-MS analysis that had markedly higher intensity in response to glucagon treatment than basal or insulin treatment (Fig. 1b, Supplementary Figs 1 and 2). Analysis of these phospho-peptides identified Ser343, Ser358, Ser379, Thr484 and Ser587 as phosphorylation sites on SIK2 (Fig. 1b). The identification of Ser342/343 was ambiguous from OrbiTrap data but data from the QTrap analysis showed that Ser343 was phosphorylated (Supplementary Fig. 1A). It should be noted that we have also identified Ser486 as a potential phosphosite (RHTL(p)SEVTNQLVVmPGAGK: (m denoting oxidized Met (Supplementary Fig. 1E)) in the phosphopeptide that contains Thr484. However, since Thr484, but not Ser486, is highly predicted as a PKA site based on consensus sequence and by Motifscan ( http://scansite.mit.edu/), we focused on the Thr484 site. Moreover, Ser379 is not conserved between human (MHSPNMRLL) and mouse (LHPPNVRLM) and was not further studied. The LC-MS-MS analysis coverage of HA–SIK2 was ~80% of the total sequence for all conditions studied (Supplementary Fig. 3). To validate the mass spectrometry-based phospho-peptide mapping results, we generated or obtained phospho-specific antibodies against four sites identified in the glucagon-treated hepatocytes (Ser343, Ser358, Thr484 and Ser587). The specificity of these antibodies was confirmed using lysates from primary hepatocytes that had been adenovirally transduced with constructs encoding WT GFP–SIK2 or the cognate phosphorylation-defective GFP–SIK2 mutants (Ser/Thr to Ala) (Supplementary Fig. 4). The effect of glucagon or insulin on recombinant SIK2 phosphorylation in primary mouse hepatocytes was then investigated using the characterized phospho-specific antibodies. In parallel experiments, endogenous SIK2 was immunoprecipitated from cell extracts, and then subjected to immunoblot analysis with the phospho-specific antibodies (Fig. 1c). In all experiments, insulin promoted Akt phosphorylation, whereas glucagon increased phosphorylation of the PKA substrate vasodilator-stimulated phosphoprotein (VASP), confirming efficacy of the hormonal treatment. Ser343, Ser358 and Thr484 phosphorylation on both the endogenous SIK2 (Fig. 1c) and adenovirally-overexpressed recombinant SIK2 proteins (Supplementary Fig. 4) was robustly increased following glucagon treatment of primary hepatocytes. In contrast, insulin had no effect on phosphorylation of any of these sites on SIK2 (Fig. 1c). Enhanced SIK2 phosphorylation was also detected with the phospho–PKA and phospho–Akt substrate antibodies following glucagon, but not following insulin treatment (Fig. 1c). Similar results were obtained in HEK293 cells where forskolin, which directly activates adenylate cyclase and stimulates cAMP production hence activating PKA, promoted SIK2 phosphorylation (both overexpressed and endogenous proteins) detected by site-specific/phospho-specific SIK2 antibodies (Supplementary Figs 5 and 6) as well as phospho–PKA and phospho–Akt substrate antibodies (Supplementary Fig. 5). Although we observed that glucagon promoted SIK2 Ser587 phosphorylation when WT GFP–SIK2 was overexpressed (Supplementary Fig. 4), we failed to observe a consistent increase in Ser587 phosphorylation in endogenous SIK2, following its immunoprecipitation in response to glucagon in primary hepatocytes. It should be noted that neither glucagon nor insulin treatment altered Thr175 phosphorylation in the activation T-loop (LKB1 site^12^) of SIK2 in hepatocytes (Fig. 1c).

Previous work reported that SIK2 Ser358 phosphorylation (as measured using a phospho–Akt substrate antibody) was increased by insulin through the PI3K–Akt2-dependent pathway in mouse liver tissue and primary hepatocytes, and that this was associated with an increase in SIK2 catalytic activity measured *in vitro*^16^. We used the SIK2 Ser358 phospho-specific antibody to analyze SIK2 phosphorylation in control or Akt2-deficient primary hepatocytes^18^ treated with insulin or a cell-permeable cAMP analogue (Bt_2_-cAMP). SIK2 was phosphorylated on Ser358 in response to Bt_2_-cAMP in both genotypes, but not in cells treated with insulin (Fig. 1d). To further clarify if there is any potential contribution of the PI3K–Akt pathway to the regulation of SIK2 Ser358 phosphorylation, immunoblot analysis was performed on lysates from primary mouse hepatocytes incubated with an Akt inhibitor (MK-2206) with or without hormones (Fig. 1e). As anticipated, MK-2206 blocked insulin-stimulated phosphorylation of Akt, but it had no influence on SIK2 Ser358 phosphorylation induced by glucagon. Incubation of hepatocytes with H-89, a selective PKA inhibitor, abolished the phosphorylation of the known PKA substrate, inositol 1,4,5-trisphosphate receptor (IP3R; ref. 19), CRTC2 dephosphorylation and also SIK2 Ser358 phosphorylation, confirming that glucagon-induced phosphorylation of Ser358 was most likely mediated through the PKA pathway (Fig. 1f).

To determine if glucagon-induced phosphorylation of SIK2 had any impact on its catalytic activity, endogenous SIK2 was immunoprecipitated from cells incubated with or without hormones, and *in vitro* kinase assays performed. As shown in Fig. 1e, SIK2 activity was unaltered by glucagon or insulin in the presence or absence of MK-2206. This was further explored by measuring the activity of recombinant SIK2 Ser/Thr to Ala mutants. We observed that all single and combined SIK2 mutants (3A; S343A/S358A/T484A or 4A; S343A/S358A/T484A/S587A) displayed comparable activity irrespective of glucagon presence in primary hepatocytes (Supplementary Fig. 7). To further validate the data obtained using primary hepatocytes in a more physiological context, immunoblot analysis of liver extracts generated from mice following 16 h/overnight fasting or 16 h fasting followed by 4 h refeeding was performed. Immunoblotting of immunoprecipitated SIK2 from these liver extracts confirmed that phosphorylation of Ser343, Ser358 and Thr484 increased following fasting but not after refeeding (Fig. 1g). Consistent with the cell-based data, immunoprecipitated SIK2 activity was the same when isolated from fasted or refed liver extracts (Fig. 1g).

Taken all together, we provide compelling evidence that cAMP/PKA-mediated signalling in response to fasting/glucagon promotes hepatic SIK2 phosphorylation at multiple sites, but in contrast there is no increase in phosphorylation of these sites with feeding/insulin. In addition, glucagon-induced SIK2 phosphorylation does not seem to influence SIK2 intrinsic catalytic activity when isolated from hepatocytes/liver, and activity is measured *in vitro* after immunoprecipitation. Since we cannot rule out the possibility that glucagon-mediated phosphorylation of SIK2 may modulate its downstream targets in intact cells without altering intrinsic activity, we overexpressed single or multi-mutants (3A or 4A) in mouse primary hepatocytes and assessed phosphorylation of CRTC2/3 and histone deacetylase 4 (HDAC4, another known SIK substrate). All the single mutants tested showed no effect on glucagon-mediated dephosphorylation. However interestingly, in SIK2 3A or 4A mutant-expressing cells glucagon-induced dephosphorylation of CRTC2/3 and HDAC4 was prevented (Fig. 1h), which was associated with a modest (~20%) inhibition of glucagon-induced glucose production (Supplementary Fig. 8A). Although this indicates that glucagon-mediated phosphorylation of SIK2 is capable of altering cellular activity by as yet unknown mechanism (s), any interpretation from overexpression studies needs to be taken with caution as 3A or 4A was overexpressed >10–20-fold above endogenous SIK2 protein (data not shown). Overexpressed SIK2 WT and 3A/4A mutants were predominantly detected in the cytoplasm (Supplementary Fig. 8B), but a relatively small amount of SIK2 may be localized in the nucleus, although this could not be precisely and quantitatively assessed. Moreover, CRTC2 localization was not altered when SIK2 3A/4A mutants were introduced (data not shown).

### SIK2 is dispensable for the regulation of hepatic gluconeogenesis

**Figure 2:**
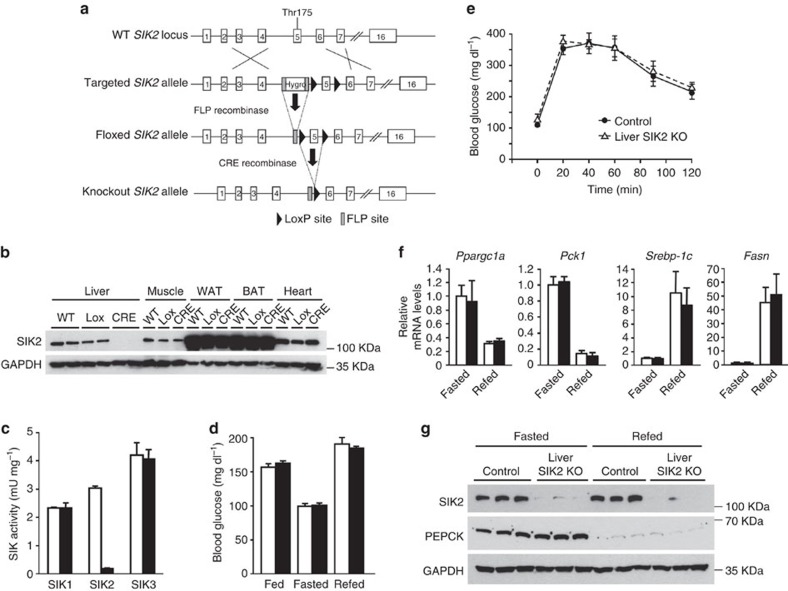
Liver-specific SIK2-knockout mice display normal glycaemia. (**a**) Diagram of the generation of liver-specific SIK2-knockout (liver SIK2 KO) mice. Exon 5 was flanked by loxP sites and a hygromycin resistance cassette flanked by FRT sites was inserted upstream from the 5′ loxP site. The hygromycin resistance cassette was excised by the expression of the FLP recombinase *in vivo*. Liver disruption of exon 5 flanked by loxP sites was achieved by crossing SIK2-floxed mice with Alfp–Cre transgenic mice. (**b**) Immunoblotting of SIK2 in liver, vastus lateralis muscle, inguinal white adipose tissue (WAT), brown adipose tissue (BAT) and heart from WT, SIK2-floxed (Lox) and liver SIK2-deficient (CRE) mice, demonstrating that the deletion was effective and liver specific. (**c**) Kinase activity of SIK1, 2 and 3 in liver extracts generated from randomly fed control (white bars) and liver SIK2-KO (black bars) mice. SIK1, 2 and 3 were immunoprecipitated with respective isoform-specific antibodies from liver extracts in triplicate and subjected to *in vitro* kinase assay. Data is presented as mean±s.d., *n*=3. (**d**) Blood glucose levels of control (white bars) and liver SIK2-KO (black bars) male mice (10-week-old) fed *ad libitum* (fed), fasted for 18 h (fasted) or refed overnight with a high-carbohydrate diet following a 24 h fast (refed). *n*=6–12 for each group. (**e**) Evaluation of blood glucose levels during an oral glucose tolerance test (3 g kg^−1^) in control and liver SIK2-KO mice (*n*=6 for each group). Values are presented as mean±s.e.m. (**f**) Relative *Ppargc1a, Pck1*, *Srebp-1c* and *Fasn* mRNA levels measured by quantitative reverse transcription–PCR in the liver of control (white bars) and liver SIK2-KO (black bars) mice fasted for 18 h or refed overnight with a high-carbohydrate diet following an 24 h fast (refed). *n*=6 for each group. All values are presented as mean±s.e.m. (**g**) Immunoblotting of SIK2, PEPCK and GAPDH in the liver of control and liver SIK2-KO mice fasted for 18 h or refed overnight with a high-carbohydrate diet following an 24 h fast (refed). Data are representative of nine mice per group.

To address whether SIK2 is necessary and plays a key role in hepatic gluconeogenesis, a liver-specific SIK2-KO mouse model was generated by Cre-LoxP recombination (Fig. 2a). SIK2^fl/fl^Cre^−/−^ (control) and liver-specific SIK2^fl/fl^Cre^+/−^ (KO) mice are indistinguishable from SIK2^+/+^ (WT) mice, displaying normal growth and no overt phenotype. Immunoblot analysis showed that SIK2 protein expression (in all tissues analyzed including liver) was comparable between the SIK2^+/+^ and SIK2^fl/fl^Cre^−/−^ mice, while a profound liver-specific depletion of SIK2 protein was observed in SIK2^fl/fl^Cre^+/−^ mice (Fig. 2b). There were no compensatory changes in SIK1 and SIK3 activity in SIK2-deficient liver (Fig. 2c).

We next examined the impact of hepatic SIK2 deficiency on blood glucose homeostasis. Liver-specific SIK2-KO mice exhibited normal blood glucose levels in randomly fed, 18-h-fasted and refed animals (Fig. 2d). Hepatic SIK2-KO mice displayed a similar blood glucose profile to control mice throughout a glucose tolerance test (Fig. 2e). Expression of key hepatic gluconeogenic and lipogenic genes was assessed in 18-h-fasted and refed animals. We observed that hepatic SIK2 deficiency had no influence on fasting- or feeding-mediated changes in the gene expression of *Pck1*, *Ppargc1a* or *Srebp-1c*, as well as *Fasn* (Fig. 2f). This was further validated by immunoblot for PEPCK protein (Fig. 2g).

**Figure 3:**
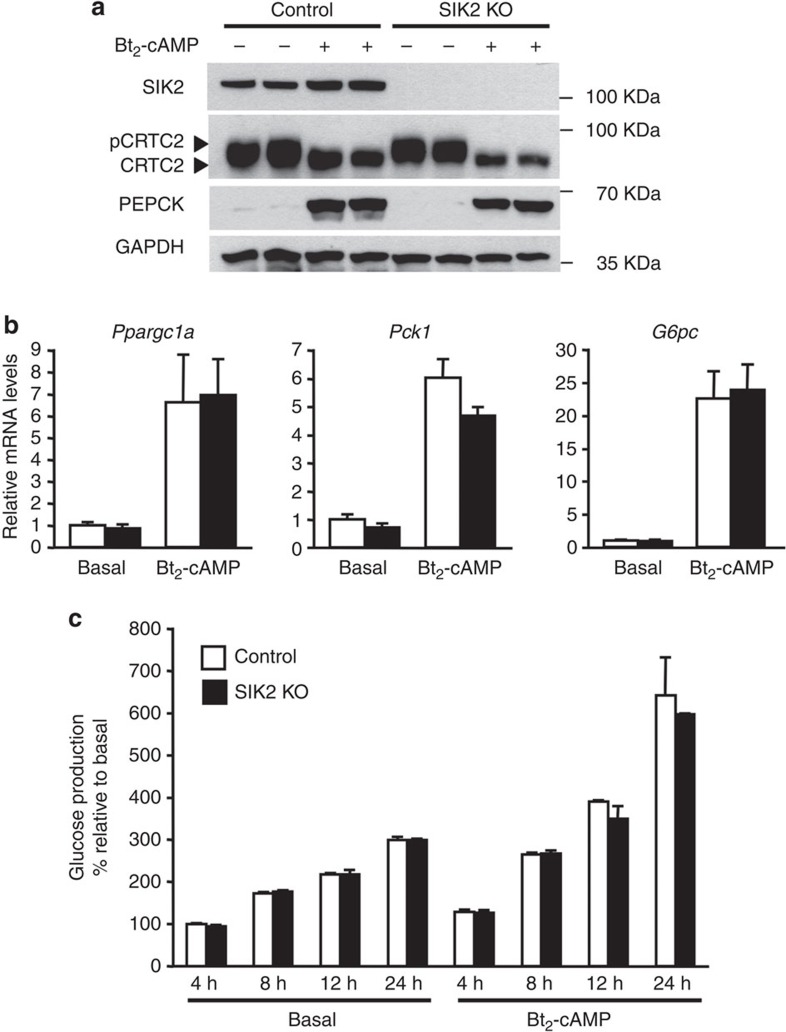
Gluconeogenesis remains unaltered in SIK2-knockout hepatocytes. (**a**) Immunoblotting of SIK2, CRTC2, PEPCK and GAPDH in primary hepatocytes isolated from control and liver SIK2-KO male mice (10-week-old) that had been treated for 8 h with or without 100 μM Bt_2_-cAMP. (**b**) Gluconeogenic gene expression measured by quantitative reverse transcription–PCR in primary hepatocytes isolated from control (white bars) and liver SIK2-KO (black bars) mice and treated for 8 h with or without 100 μM Bt_2_-cAMP. Transcript levels in control cells were assigned an arbitrary value of 1.0 for comparison. (**c**) Glucose production was measured in the culture medium of primary hepatocytes isolated from control and liver SIK2-KO mice 4, 8, 12 or 24 h after treatment with or without 100 μM Bt_2_-cAMP as described in Methods. Glucose production was normalized to protein content and expressed as a percentage of glucose production by control hepatocytes incubated for 4 h in the absence of Bt_2_-cAMP. All values are presented as mean±s.e.m., *n*=3.

We confirmed this *in vivo* data by examining the effect of SIK2 deficiency on gluconeogenesis in primary hepatocytes. We first assessed cAMP–PKA-mediated changes in CRTC2 dephosphorylation and gluconeogenic gene expression in control and SIK2-KO hepatocytes. Treatment of hepatocytes with Bt_2_-cAMP caused a robust downward gel-shift/dephosphorylation of CRTC2 (Fig. 3a), and increases in messenger RNA (mRNA) expression of *Ppargc1a*, *Pck1* and *G6pc*, with no difference between control and SIK2-KO hepatocytes (Fig. 3b). In addition, induction of PEPCK protein expression was comparable between control and SIK2-KO hepatocytes (Fig. 3a). Consistent with these results and also with data from *in vivo* experiments (Fig. 2d,f and g), basal and Bt_2_-cAMP-stimulated glucose production (4–24 h) in primary hepatocytes was not altered by genetic deletion of SIK2 (Fig. 3c).

**Figure 4:**
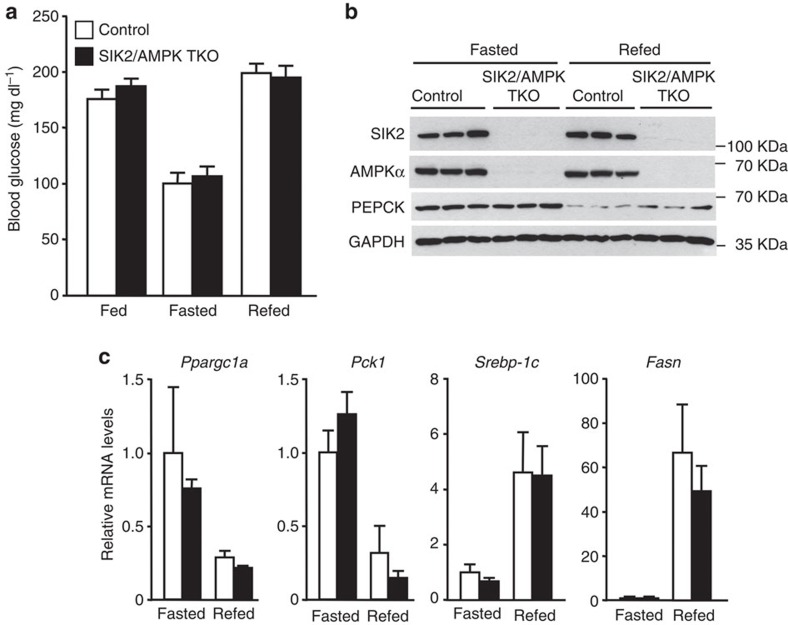
Liver SIK2/AMPKα1/AMPKα2 triple knockout mice display normal glycaemia. (**a**) Blood glucose levels of control (white bars) and liver SIK2/AMPKα1/AMPKα2 triple KO (black bars) male mice (10-week-old) fed *ad libitum* (fed), fasted for 18 h (fasted) or refed overnight with a high-carbohydrate diet following a 24 h fast (refed). *n*=5–8 for each group. (**b**) Immunoblotting of SIK2, AMPKα, PEPCK and GAPDH in the liver of control and liver SIK2/AMPKα1/AMPKα2 triple KO mice (SIK2/AMPK TKO) fasted for 18 h or refed overnight with a high-carbohydrate diet following a 24 h fast (refed). Data are representative of six mice per group. (**c**) Relative *Ppargc1a, Pck1, Srebp-1c* and *Fasn* mRNA levels measured by reverse transcription–PCR in the liver of control (white bars) and liver SIK2/AMPK TKO (black bars) mice fasted for 18 h or refed overnight with a high-carbohydrate diet following a 24 h fast (refed). *n*=4–7 for each group. All values are presented as mean±s.e.m.

**Figure 5:**
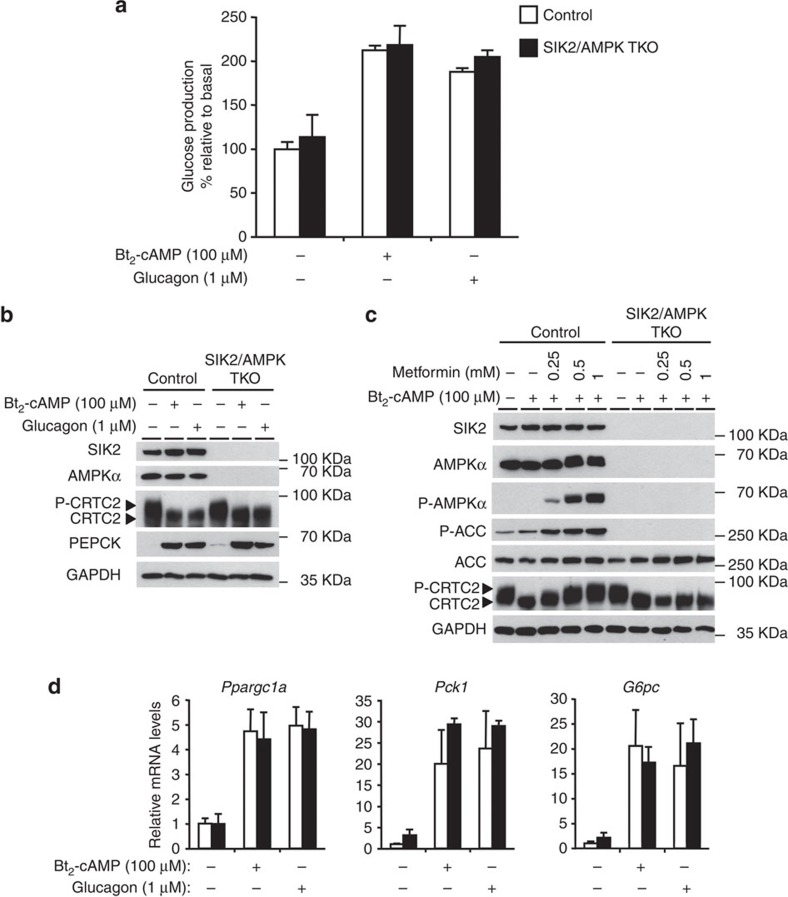
Gluconeogenesis is unaltered in SIK2/AMPKα1/AMPKα2 triple knockout hepatocytes. (**a**) Glucose production was assessed by measuring glucose in the culture medium of primary hepatocytes isolated from control and liver SIK2/AMPKα1/AMPKα2 triple KO (SIK2/AMPK TKO) male mice (10-week-old) 8 h after treatment with or without 100 μM Bt_2_-cAMP or 1 μM glucagon. Glucose production was normalized to protein content and expressed as a percentage of glucose production by control hepatocytes incubated in the absence of Bt_2_-cAMP or glucagon. (**b**) Immunoblotting of SIK2, AMPKα, phospho (P)-CRTC2/CRTC2, PEPCK and GAPDH in primary hepatocytes isolated from control and liver SIK2/AMPKα1/AMPKα2 triple KO (SIK2/AMPK TKO) mice and treated for 8 h with or without 100 μM Bt_2_-cAMP or 1 μM glucagon. (**c**) Immunoblotting of SIK2, AMPKα, P-AMPKα, ACC, P-ACC, P-CRTC2/CRTC2 and GAPDH in primary hepatocytes isolated from control and liver SIK2/AMPKα1/AMPKα2 triple KO (SIK2/AMPK TKO) mice and treated for 8 h with or without 100 μM Bt_2_-cAMP and with or without 0.25, 0.5 or 1 mM metformin as indicated. (**d**) Gluconeogenic gene expression was measured by quantitative reverse transcription–PCR in primary hepatocytes isolated from control (white bars) and liver SIK2/AMPK TKO (black bars) mice and treated for 8 h with or without 100 μM Bt_2_-cAMP or 1 μM glucagon. Transcript levels in control cells were assigned an arbitrary value of 1.0 for comparison. All values are presented as mean±s.e.m. Results are representative of at least three independent experiments.

Given that SIK2 and AMPK are able to phosphorylate CRTC2 on a common site (Ser171; refs 11, 20, 21), we wanted to check if AMPK might compensate for the lack of SIK2 and vice versa. For this purpose, we generated mice lacking SIK2, AMPKα1 and AMPKα2 simultaneously and specifically in the liver. Triple deletion of hepatic AMPKα1/α2 and SIK2 did not affect blood glucose levels in randomly fed, 18-h-fasted and refed mice (Fig. 4a). Furthermore, expression of key hepatic gluconeogenic (*Ppargc1a, Pck1*) and lipogenic (*Srebp-1c, Fasn*) and hepatic PEPCK protein levels were similar between control and triple KO (TKO) mice (Fig. 4b,c). Consistent with these observations, basal and Bt_2_-cAMP- or glucagon-induced glucose production were comparable between control and the triple AMPKα1/α2/SIK2-deficient hepatocytes (Fig. 5a). CRTC2 gel mobility in unstimulated or stimulated (Bt_2_-AMP or glucagon) conditions was comparable between the triple AMPKα1/α2/SIK2-deficient and control hepatocytes (Fig. 5b). In contrast, phosphorylation of CRTC2 and acetyl-CoA carboxylase (ACC) in response to metformin was blunted in TKO hepatocytes (Fig. 5c) as previously observed in AMPK-deficient hepatocytes^4^. Gluconeogenic gene (*Ppargc1a, Pck1 and G6pc*) expression was comparable between control and the TKO hepatocytes in unstimulated or stimulated (Bt_2_-AMP or glucagon) conditions (Fig. 5d).

These results highlight that deletion of AMPK or SIK2, alone or in combination, are not sufficient to mimic the alteration in the regulation of gluconeogenesis (Figs 2, 3, 4, 5) observed in LKB1-deficient liver and hepatocytes^4^,^11^.

### Inhibition of SIKs promotes glucose production in primary hepatocytes

The previously described experiments suggest that SIK2 is dispensable for control of hepatic gluconeogenesis, perhaps because loss of SIK2 can be compensated by other SIK isoforms (but not by AMPK). To fully delineate the role of SIKs in hepatic gluconeogenesis, it would be necessary to ablate the activity of all SIK isoforms in the liver. We took advantage of recently identified pan-SIK inhibitors whose potency and specificity have been well-characterized *in vitro* and in intact cells (macrophages)^22^. Of note, they are specific to SIK isoforms and require >100-fold concentration to inhibit AMPK and other AMPK-related kinases and LKB1 in cell-free assays^22^.

**Figure 6:**
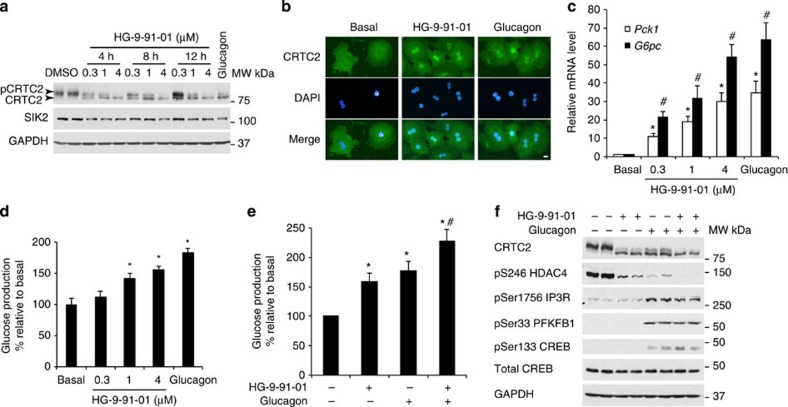
The SIK inhibitor HG-9-91-01 increases hepatic gluconeogenesis. (**a**) Primary hepatocytes (8–12-week-old male C57BL/6) were treated with the indicated concentrations of HG-9-91-01 for 4, 8 or 12 h prior to cell lysis. Lysates were subjected to immunoblotting with the indicated antibodies. Primary hepatocytes were also treated with 0.1 μM glucagon for 12 h and included in the experiment as a positive control. (**b**) Primary hepatocytes were treated with 4 μM HG-9-91-01 for 1 h and 0.1 μM glucagon for 10 min. Subsequently cells were fixed with 3.7% paraformaldehyde and immunostained using a CRTC2 antibody and stained with 4′,6-diamidino-2-phenylindole (DAPI) for nuclear visualization. Images were captured with a fluorescence microscope (Nikon Eclipse Ti) using Nikon NIS-Elements BR 3.1 software. Scale bar is 10 μm in length. (**c**) Relative mRNA levels of *Pck1* and *G6pc* were measured from primary hepatocytes using quantitative reverse transcription–PCR after an 8 h treatment with the indicated amount of HG-9-91-01 or 0.1 μM glucagon. The mRNA levels were presented as fold increase from non-treated hepatocytes (basal) and are presented as mean±s.d., *n*=3. *P<0.01 basal versus each treatment for *Pck1* mRNA, # *P*<0.01 basal versus each treatment for *G6pc* mRNA. (**d**) Cell culture medium (12 h treatment) from the same experiment (**c**) was used to analyze glucose production. Glucose production was normalized to total cellular protein content and presented as percentage of glucose production by hepatocytes without treatment (basal). Glucagon treatment (0.1 μM) for 12 h was used as a positive control. Data is presented as mean±s.d., *n*=6. *P<0.01 basal versus 1 μM HG-9-91-01, 4 μM HG-9-91-01 or glucagon. (**e**) Glucose production was measured from primary hepatocytes that were treated with 4 μM HG-9-91-01 and/or 0.1 μM glucagon for 8 h. Glucose production was normalized to total protein content and presented as percentage of glucose production by hepatocytes without treatment (basal). Data is presented as mean±s.d., *n*=6. *P<0.01 basal versus HG-9-91-01 or glucagon, ^#^*P*<0.05 HG-9-91-01 or glucagon versus HG-9-91-01 plus glucagon. (**f**) Primary hepatocytes were treated with 4 μM HG-9-91-01 for 8 h and/or 0.1 μM glucagon prior to cell lysis. Lysates were immunoblotted with the indicated antibodies.

We first performed a dose-response (0.1–4 μM) and time-course (4–12 h) study to determine the concentration of SIK inhibitor (HG-9-91-01) that is required for a robust and sufficient suppression of SIK activity in mouse primary hepatocytes. We confirmed that 4 μM is the saturating concentration required for promoting CRTC2 dephosphorylation and glucose production in mouse primary hepatocytes (data not shown). We assessed SIK activity by monitoring the phosphorylation state of CRTC2 as indicated by band-mobility shift and observed that HG-9-91-01 caused a dose-dependent increase in a faster migrating form of CRTC2 (dephosphorylation) with a reciprocal reduction in the slower migrating phosphorylated form (Fig. 6a). CRTC2 dephosphorylation in primary hepatocytes was more pronounced following exposure to 4 μM HG-9-91-01 than that generated by 0.1 μM glucagon treatment.

It has been reported that in unstimulated hepatocytes, CRTC2 is highly phosphorylated and mostly localized in the cytoplasm. Upon treatment with glucagon or stimuli that induce cellular cAMP elevation, CRTC2 is dephosphorylated and translocates into the nucleus^11^,^21^. Therefore, we used immunofluorescence to examine if HG-9-91-01-induced dephosphorylation of endogenous CRTC2 leads to its nuclear enrichment. As previously reported, we found that CRTC2 is predominantly localized in the cytoplasm of unstimulated cells. HG-9-91-01 treatment caused marked enrichment of CRTC2 in the nucleus, comparable to the translocation in response to glucagon (Fig. 6b).

We next examined the effects of HG-9-91-01 on hepatic gluconeogenesis by measuring gluconeogenic gene expression and glucose production. HG-9-91-01 treatment dose dependently increased mRNA expression of *Pck1* and *G6pc* and that effect was similar in cells treated with 4 μM HG-9-91-01 or 0.1 μM glucagon (Fig. 6c). Consistent with this observation, there was also a dose-dependent increase in glucose production following HG-9-91-01 treatment (Fig. 6d). The structurally related SIK inhibitor KIN112 (10 μM; ref. 22) modestly, but significantly, increased glucose production, CRTC2 and CRTC3 dephosphorylation and PEPCK protein expression in mouse primary hepatocytes (Supplementary Fig. 9).

Given the observation that glucagon and HG-9-91-01 both promoted CRTC2 dephosphorylation, gluconeogenic gene expression and glucose production in primary hepatocytes, we hypothesized that glucagon-induced gluconeogenesis is at least in part SIK dependent. Thus, we speculated that combined treatment (HG-9-91-01 and glucagon) would not display an additive increase in glucose production compared with individual treatments. Contrary to our hypothesis, there was a significant additive increase in glucose production when the two agents were given to hepatocytes simultaneously (Fig. 6e), which was accompanied by more pronounced dephosphorylation of CRTC2 and HDAC4 than following individual treatments (Fig. 6f).

The possible mechanism behind this additive effect was investigated by measuring the phosphorylation of glucagon-regulated signalling molecules proposed to be involved in hepatic gluconeogenesis. In line with the observations shown in previous studies, glucagon-induced phosphorylation of CREB, 6-phosphofructo-2-kinase/fructose-2,6-biphosphatase 1 (PFKFB1) and IP3R (Fig. 6f), which are known PKA substrates recently implicated in the control of hepatic gluconeogenesis^10^,^19^,^23^. In contrast, HG-9-91-01 had no effect on the phosphorylation of these proteins. Combined treatment with HG-9-91-01 and glucagon did not further enhance phosphorylation of these proteins (CREB, PFKFB1 and IP3R) compared with glucagon alone. However, it did have an additive effect on the dephosphorylation of CRTC2 (Fig. 6f), which could explain the additive effect on glucose production.

### HG-9-91-01-induced glucose production is dependent on LKB1 but not AMPK

**Figure 7:**
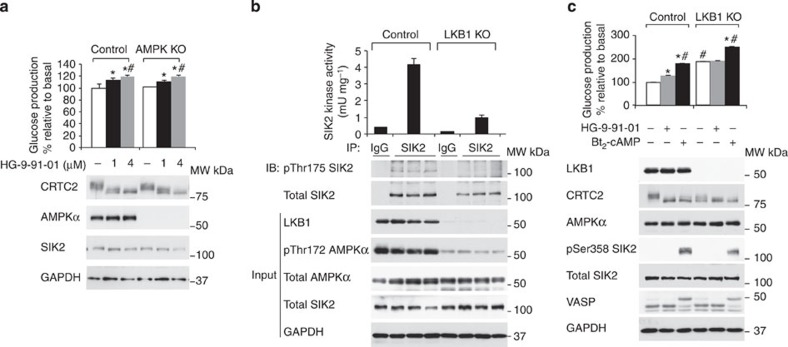
Effect of HG-9-91-01 in AMPK- or LKB1-knockout primary hepatocytes. (**a**) Primary hepatocytes from control (AMPKα1^lox/lox^α2^lox/lox^) and liver-specific AMPKα1/α2 knockout (AMPK KO) male mice (10-week-old) were treated with the indicated amount of HG-9-91-01 for 8 h prior to cell lysis. Immunoblot analysis was conducted with the indicated antibodies and glucose was measured in the culture medium. Glucose production was normalized to total protein content and presented as percentage of glucose production by non-treated hepatocytes. Data is presented as mean±s.d., *n*=3. *P<0.01 basal versus 1 μM HG-9-91-01 treatment in both genotypes and #*P*<0.001 basal versus 4 μM HG-9-91-01 treatment in both genotypes. (**b**) Endogenous SIK2 was immunoprecipitated from 1 mg liver extracts of control (LKB1^lox/lox^) or liver-specific LKB1 knockout (LKB1 KO) mice using SIK2 antibody or pre-immune immunoglobulin G (IgG) and subjected to either *in vitro* kinase assay or immunoblot analysis. Pre-immune IgG was used as negative control. Data is presented as mean±s.d., *n*=3. Same liver extracts (40μg) were included in immunoblot analysis as input. (**c**) Glucose production from primary hepatocytes of control (LKB1^lox/lox^) or liver-specific LKB1 knockout (LKB1 KO) mice was measured following 4 μM HG-9-91-01 or 100 μM Bt_2_-cAMP treatment for 8 h. Lysates from the same experiment were immunoblotted with the indicated antibodies. Data is presented as mean±s.d., *n*=3. *P<0.01 basal versus each treatment in control and basal versus Bt_2_-cAMP in LKB1 KO, ^#^*P*<0.01 basal control versus basal LKB1 KO, Bt_2_-cAMP control versus Bt_2_-cAMP LKB1 KO.

LKB1-dependent kinases, including AMPK and SIKs^13^, are reported to phosphorylate CRTC2 and class IIa HDACs, and thus proposed to regulate hepatic gluconeogenesis^20^,^24^. Despite the high homology of the kinase domain between AMPK and SIKs, HG-9-91-01 has more than a 100-fold greater potency against SIKs than AMPK in a cell-free assay^22^. However, the effect of HG-9-91-01 on AMPK in intact cells has not been examined. Therefore it remained plausible that at least part of the effect of this compound on hepatic gluconeogenesis could be mediated through AMPK. To this end, the effects of HG-9-91-01 on hepatic gluconeogenesis were investigated in primary hepatocytes lacking both AMPKα catalytic isoforms (α1/α2; ref. 4). Both control and AMPKα1/α2 KO hepatocytes showed similar basal as well as HG-9-91-01-induced CRTC2 dephosphorylation and glucose production (Fig. 7a). These results suggested that the ability of HG-9-91-01 to promote hepatic glucose production does not involve AMPK.

Catalytic activity of SIK requires LKB1-dependent T-loop phosphorylation and thus SIK activity in LKB1-deficient HeLa cells and mouse embryonic fibroblasts is profoundly reduced^12^. We next examined the effect of HG-9-91-01 on glucose homeostasis in mouse primary hepatocytes, isolated from a liver-specific LKB1 KO mouse, to establish the dependency for LKB1-induced SIK activity. Deletion of LKB1 was confirmed by immunoblotting of liver tissue extracts derived from LKB1 KO compared with control animals (Fig. 7b). LKB1 protein expression was nearly ablated and T-loop phosphorylation of AMPKα was robustly reduced in LKB1-deficient liver as shown previously^4^.

SIK2 T-loop phosphorylation and activity were assessed after immunoprecipitation by immunoblotting and *in vitro* kinase assay, respectively. We observed that SIK2 protein expression was comparable between control and LKB1-deficient liver, while T-loop (Thr175) phosphorylation was undetectable in liver extracts derived from liver LKB1 KO mice (Fig. 7b). Consistent with this observation, SIK2 activity was profoundly reduced in LKB1 KO liver. Residual expression of LKB1 and SIK2 activity detected in LKB1-deficient liver could be due to non-hepatic cells (for example, Kupffer cells and fibroblasts) present in whole liver tissue.

Next, we tested the response of primary hepatocytes isolated from control and liver LKB1 KO mice to either HG-9-91-01 or Bt_2_-cAMP. As described in our previous work^4^, we observed that there was much lower phosphorylation of CRTC2 in LKB1-deficient hepatocytes compared with control, in unstimulated cells, which was associated with a significantly higher glucose production in LKB1-deficient hepatocytes (Fig. 7c). In control hepatocytes HG-9-91-01 treatment again caused a robust downward band shift of total CRTC2 and a significant increase in glucose production. In contrast, HG-9-91-01 resulted in only a modest increase in band shift with no increase in glucose production in LKB1 KO hepatocytes (Fig. 7c). These data indicate a key functional role for the LKB1–SIK system as a ‘repressor’ of hepatic gluconeogenesis. Interestingly, Bt_2_-cAMP was able to promote a further band shift of CRTC2, and significantly stimulate glucose production in these cells.

### A drug-resistant SIK2 mutant abolishes HG-9-91-01-induced gluconeogenesis

**Figure 8:**
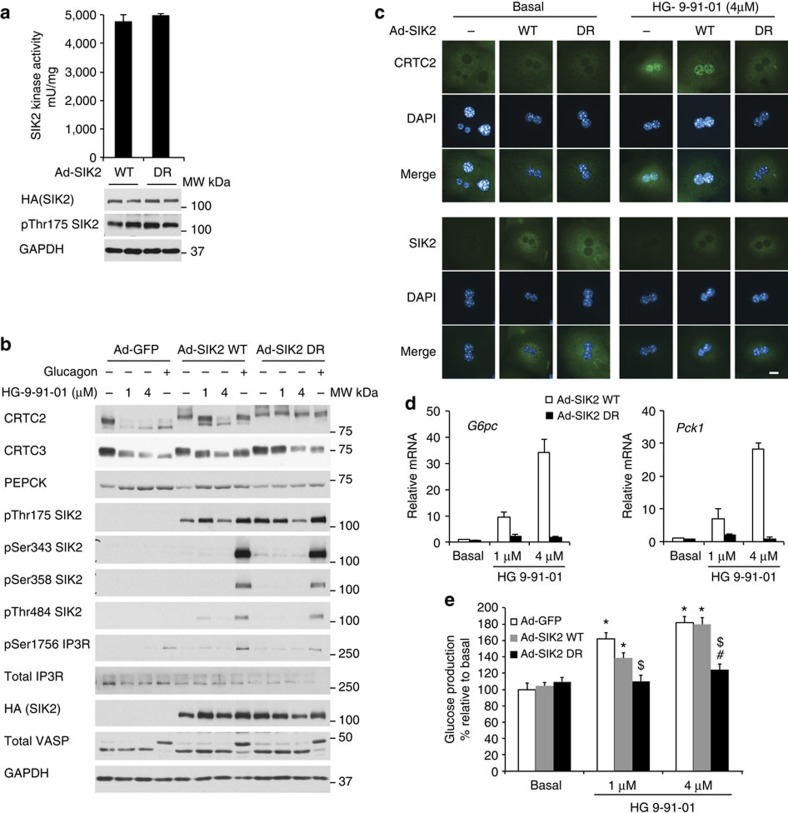
An SIK2 drug-resistant mutant prevented the effects of HG-9-91-01. (**a**) HA-tagged SIK2 wild-type (WT) or a T96Q drug-resistant (DR) mutant were expressed in primary mouse hepatocytes (from 8–12-week-old male C57BL/6) for 16 h using adenoviral vectors (1:5 MOI) prior to cell lysis. HA–SIK2 was immunoprecipitated from 100 μg lysate and kinase activity was assayed. Data is presented as mean±s.d., *n*=3. Lysates were also immunoblotted with the indicated antibodies. (**b**) Primary hepatocytes were transduced for 24 h (1:5 MOI) with HA–SIK2 wild-type (Ad-SIK2 WT), HA–SIK2 T96Q drug-resistant mutant (Ad-SIK2 DR) or Ad-GFP (as infection control) adenoviruses prior to cell lysis. The cells were treated for the last 12 h with 1 or 4 μM HG-9-91-01 or 0.1 μM glucagon and lysates were subjected to immunoblotting with the indicated antibodies. (**c**) Primary hepatocytes were infected (1:2 MOI) with HA–SIK2 adenovirus (Ad-SIK2) WT or DR mutant for 16 h followed by 4 μM HG-9-91-01 treatment for 1 h before fixing the cells. The cells were immunostained with the indicated antibodies. 4′,6-diamidino-2-phenylindole (DAPI) was used for nuclear staining. Images were captured with a fluorescence microscope (Nikon Eclipse Ti) using Nikon NIS-Elements BR 3.1 software. Scale bar is 10 μm in length. (**d**) Gluconeogenic gene expression was measured by quantitative reverse transcription–PCR in primary hepatocytes under the same conditions as in **b** other than glucagon treatment. (**e**) The cell culture medium from the experiment in **b** was used to measure glucose production. Glucose production was normalized to total protein content and presented relative to hepatocytes without treatment (basal). Data is presented as mean±s.d., *n*=6. *P<0.01 basal versus each treatment in Ad-GFP and in Ad-SIK2 WT group, basal versus glucagon in Ad-SIK2 DR group. ^$^*P*<0.01 HG-9-91-01 treatment in SIK2 WT versus SIK2 DR and ^#^*P*<0.05 basal versus HG-9-91-01 treatment in Ad-SIK2 DR.

A powerful approach to establish the observed effect of a compound, that inhibits a kinase of interest is not an ‘off-target’ effect, is to demonstrate that the inhibitor action is lost when a drug-resistant mutant kinase is expressed in place of the WT kinase. Clark *et al.*^22^ recently reported that the mutation of the gatekeeper (a particular amino acid located towards the back of the ATP pocket, which is a crucial determinant of kinase inhibitor selectivity) threonine (SIK1 Thr103, SIK2 Thr96 and SIK3 Thr146) to glutamine made SIK isoforms 100–1000-fold less sensitive to HG-9-91-01 *in vitro* and the mutant proteins are resistant to HG-9-91-01 when overexpressed in HEK293 cells (Supplementary Fig. 10). Since SIK2 is relatively highly expressed in the liver, the SIK2 drug-resistant mutant (T96Q) was introduced in hepatocytes using an adenoviral expression construct, and its expression and function were studied in comparison with WT SIK2 (Fig. 8a). Importantly, expression and kinase activity as well as T-loop phosphorylation (Thr175) were similar between WT and the drug-resistant mutant of SIK2 (Fig. 8a). As anticipated, glucagon caused comparable phosphorylation of PKA substrates (IP3R and VASP) in all conditions and induced SIK2 phosphorylation on Ser343, Ser358 and Thr484 in a similar manner in SIK2 WT- and drug-resistant mutant-expressing cells (Fig. 8b).

We then sought to determine if expression of the drug-resistant SIK2 mutant would abolish the downstream effects of HG-9-91-01 and glucagon in hepatocytes. We infected mouse primary hepatocytes with an adenovirus encoding WT HA–SIK2, drug-resistant HA–SIK2 mutant (T96Q) or GFP alone (control) and the cells were then left untreated, treated with HG-9-91-01 (1 or 4μM) or with glucagon (0.1 μM) (Fig. 8b). In untreated hepatocytes, expression of SIK2 WT and drug-resistant mutant was similar and both conditions caused modestly higher phosphorylation of CRTC2/3 compared with the GFP control. Consistent with the previous experiments (for example, Fig. 6a), HG-9-91-01 treatment promoted a robust dephosphorylation of both CRTC2 and CRTC3 in control GFP-expressing cells and also WT SIK2-expressing cells. The dephosphorylation of CRTC2/3 was abrogated following HG-9-91-01 treatment of the cells expressing the drug-resistant SIK2 mutant (Fig. 8b). Strikingly HG-9-91-01-induced nuclear enrichment of CRTC2 was abolished in drug-resistant mutant-expressing hepatocytes (Fig. 8c). Wild-type and drug-resistant mutant SIK2 both appeared to localize in the cytosol and remained unaltered by HG-9-91-01 treatment in hepatocytes. HG-9-91-01-stimulated glucose production and induction of PEPCK protein expression, as well as mRNA expression of *Pck1* and *G6pc* was observed in control GFP- and WT SIK2-expressing cells but all actions were lost in the hepatocytes expressing the drug-resistant SIK2 mutant (Fig. 8b,d,e), demonstrating that SIK2 mediates these actions of the drug.

## Discussion

Previous work by Dentin *et al.*^16^ reported that SIK2 plays an important role in the control of insulin/feeding-mediated suppression of hepatic gluconeogenesis through phosphorylation/activation of SIK2 downstream of the PI3K–Akt signalling pathway. In sharp contrast to this mechanism proposed by Dentin *et al.*^16^, here we provide compelling opposing evidence that insulin does not promote phosphorylation and activation of SIK2 in the liver, and go on to show that liver-specific ablation of SIK2 alone has no impact on fasting- and feeding-mediated changes in gluconeogenic gene expression and blood glucose levels *in vivo*.

We believe that the identification of Ser358 as a potential Akt phosphorylation site in SIK2 in the previous work^16^ was indirect and not robust. For example, it was initially identified based only on the fact that the sequence around Ser358 conforms to the minimum kinase consensus motif for Akt phosphorylation, and the evidence that phosphorylation of endogenous SIK2 at Ser358 actually occurs in cells exposed to insulin was supported only by the use of a generic phospho–Akt substrate-motif antibody^16^. Work in cell-free assays and experiments with recombinant WT and a S358A mutant of SIK2 expressed in HEK293 cells provided evidence that Akt could phosphorylate wild type, but not S358A SIK2.

The key finding that insulin promoted SIK2 phosphorylation detected by the phospho–Akt substrate antibody^16^ was not reproduced in our previous^17^ or current work, despite performing multiple experiments in various cell systems (hepatocytes, adipocytes and HEK293 cells (Supplementary Figs 5 and 6)). Most importantly, our SIK2 phosphorylation analyses using either unbiased mass spectrometry or site-specific phospho-antibodies (as well as the phospho–Akt/PKA substrate-motif antibodies), found no evidence that Ser358 phosphorylation was induced by insulin/feeding, yet both approaches were able to identify this as a major modification induced by glucagon/fasting. It is worth noting that we confirmed that the phospho–Akt substrate-motif (Arg–Xaa–Arg–Xaa–Xaa–*p*Ser/Thr, Xaa, any amino acid) and the phospho–PKA substrate-motif (Arg–Arg–Xaa–*p*Ser/T) antibodies both detect multiple phosphorylation sites on SIK2 due to their overlapping epitopes. To eliminate glucagon-induced phosphorylation detected using these antibodies we had to simultaneously mutate three (3A; Ser343, Ser358 and Thr484) or four (3A and Ser587) sites on SIK2 (Supplementary Fig. 4). This seriously questions their utility in precise phosphorylation-site mapping experiments.

In line with what we previously demonstrated in adipocytes treated with cAMP-inducing agents^17^, we found that SIK2 becomes phosphorylated at multiple sites in response to glucagon/fasting, although the T-loop site that is phosphorylated by LKB1 remains unchanged. Importantly, the intrinsic activity of SIK2, isolated from liver cell/tissue extracts and assayed *in vitro*, was not different in samples following fasting/glucagon to that measured from fed/insulin-treated or untreated tissue/cells. Consistent with this, mutations of any or all (4A; four Ser/Thr residues to Ala) of the glucagon-induced phosphorylation sites had no effect on SIK2 activity and did not alter phosphorylation in the activation T-loop Thr175 site (Supplementary Fig. 7). These results are in line with what we previously demonstrated in adipocytes, and suggest that phosphorylation of Ser343, Ser358, Thr484 and Ser587 (individually or all together) does not in itself modulate intrinsic kinase activity. Instead, it appears that phosphorylation of Thr175 by LKB1 maintains constant SIK2 intrinsic kinase activity in hepatocytes, irrespective of the presence of hormones. However, we cannot rule out the possibility that isolation of SIK2 by immunoprecipitation separates the kinase from a co-factor (s) or interacting protein(s) that modulate activity of SIK2, and the *in vitro* kinase assay may not truly reflect the *in vivo*/cellular activity status of SIK2. Given that cAMP/PKA-mediated phosphorylation promotes binding to 14-3-3 adaptor proteins^17^, it is possible that glucagon-mediated phosphorylation of SIK2 alters its cellular localization and/or protein-protein interactions and consequently modulates signalling. One interesting observation was that when we overexpressed individual or combination (3A or 4A) SIK2 mutants in mouse primary hepatocytes, 3A- or 4A-SIK2-expressing cells did not display glucagon-mediated dephosphorylation of CRTC2/3. This possibly indicates that glucagon-mediated phosphorylation plays an ‘inhibitory role’ on SIK activity *in vivo*. However, this has to be cautiously interpreted as the 3A or 4A mutants were overexpressed >10–20-fold above endogenous SIK2 protein and thus may have acted as dominant negative molecules. Monitoring cellular SIK activity in response to glucagon is challenging one approach would be to generate a fluorescent reporter^25^ containing a SIK-targeting sequence and measure kinetic changes in *de novo* SIK activity in response to glucagon in hepatocytes.

Nonetheless, collectively the current results do not support the proposed model that insulin-mediated suppression of hepatic gluconeogenesis involves phosphorylation and activation of SIK2. It can be noted that the same group that published the insulin–Akt regulation of SIK2 (ref. 16) recently proposed a new hypothesis/pathway that insulin/feeding switches off hepatic gluconeogenesis through Akt-mediated phosphorylation of IP3R (Ser2682). IP3R phosphorylation promotes CRTC2 phosphorylation and inhibition of gluconeogenic gene expression^19^, providing an alternative SIK2-independent pathway from the insulin receptor to reduced gluconeogenesis. Indeed, there are multiple pathways known to turn off gluconeogenesis by insulin including Akt2-dependent regulation of FoxO1 and redirection of G6P to glycogen^1^,^18^.

In the current study, we used pan-SIK inhibitors together with an inhibitor-resistant SIK mutant to reveal that acute and selective pharmacological inhibition of SIK (s) significantly increases hepatic gluconeogenesis in primary hepatocytes. On the other hand, we also provided genetic evidence that ablation of SIK2 in the liver does not affect hormone-mediated changes in gluconeogenesis and we confirmed that there were no compensatory changes in the activity of other SIK isoforms (SIK1/3) in SIK2-deficient liver. These results suggest that SIK2 has no significant role in the control of hepatic gluconeogenesis, or alternatively, there is a degree of redundancy among the SIK isoforms that does not require upregulation of SIK protein. To test these hypotheses we performed short hairpin RNA interference -mediated knockdown of the SIK isoforms (singly and in combination) using adenovirus infection of SIK2-null mouse primary hepatocytes. However, we could achieve only ~80% knockdown of SIK1 or 3, which did not result in significant changes in CRTC2 phosphorylation and the amount of adenovirus (scramble control) required for dual (SIK1&3) knockdown itself affected glucose production (~30% reduction in unstimulated cells; data not shown). Therefore, this approach could not provide compelling evidence to conclude whether there is a dominant SIK isoform or whether SIK isoforms play a redundant role in the control of hepatic gluconeogenesis. As a next step, it would be interesting to generate and analyze liver-specific SIK1 or SIK3, and if viable, double (SIK1/3) or TKO (SIK1/2/3) mouse models.

A key finding of this study is that the cAMP–PKA pathway and SIKs appear to regulate CRTCs and gluconeogenesis at least partially through distinct mechanisms. This notion is based on the following: (1) Bt_2_-cAMP, but not the SIK inhibitor promoted glucose production in LKB1-deficient hepatocytes (Fig. 7c), suggesting that SIK activity is not required for cAMP regulation of glucose production and, (2) co-incubation with HG-9-91-01 and glucagon resulted in an additive effect on glucose production. In line with these findings, glucagon promotes mobilization of Ca^2+^ through phosphorylation of IP3R (Ser1756), which in turn activates calcineurin (also known as protein phosphatase 2B) leading to dephosphorylation and nuclear translocation of CRTCs, and a resultant activation of CREB-regulated gluconeogenic genes in hepatocytes^19^. A calcineurin inhibitor (cyclosporin A) blocked glucagon-induced dephosphorylation of CRTC2 in concert with gluconeogenic gene promoter activity^19^; thus, it would be interesting to determine if HG-9-91-01-induced glucose production is sensitive to cyclosporin A. In addition to this mechanism it has also been proposed that glucagon increases hepatic glucose production by regulating Ca^2+^-CaMKII-mediated FoxO1 nuclear localization^26^. Moreover, although CRTC2 has been proposed as a key regulator for gluconeogenic gene expression and glucose production, CRTC2 is dispensable in glucagon-mediated glucose production^27^ and we also observed that HG-9-91-01 stimulates glucose production in CRTC2-deficient primary hepatocytes (Supplementary Fig. 11). All in all, the precise signalling pathways that mediate glucagon-induced hepatic gluconeogenesis are still not fully understood, but most likely involve the key second messengers, cAMP and calcium ions, and multiple downstream effectors such as kinases (for example, PKA, CaMKII) and phosphatases (for example, calcineurin) with subsequent regulation of multiple cytosolic PKA targets (for example, 6-phosphofructo-2-kinase, IP3R) and transcription factors/co-activators (for example, CRTCs, FoxOs, HNF-4α, PGC-1α, histone acetylase/deacetylases) that directly or indirectly regulate gluconeogenic gene expression^1^,^10^,^19^,^24^,^26^.

**Figure 9:**
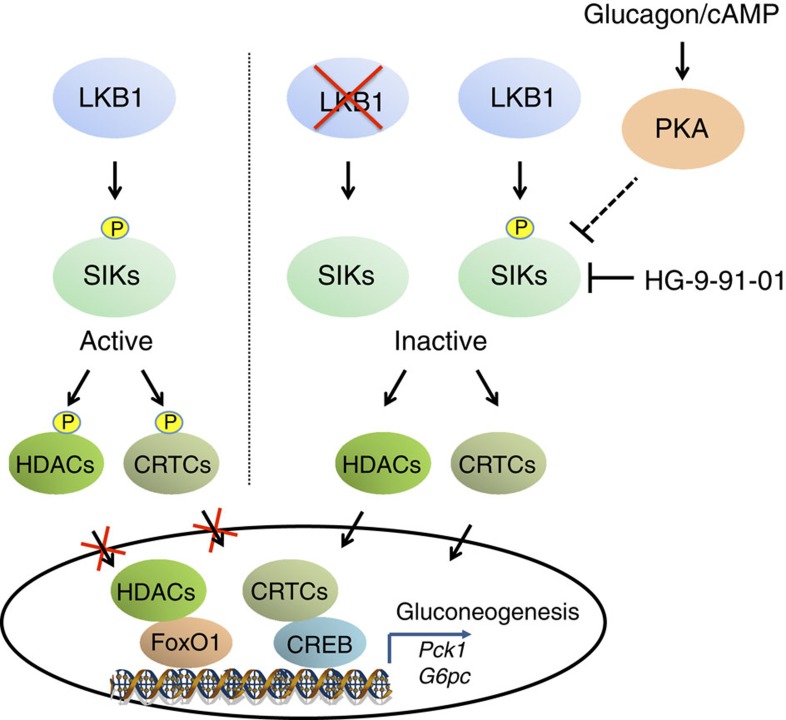
Schematic diagram showing LKB1–SIK pathway in the control of hepatic gluconeogenesis. Activity status of SIKs influences phosphorylation of transcription co-activators such as HDACs and CRTCs their nuclear-cytoplasm shuttling and gluconeogenic gene expressions in the liver.

In summary, we provide evidence that SIK isoforms significantly contribute to suppression of hepatic gluconeogenesis by regulating downstream transcription factors/co-activators and that glucagon (but not insulin) can regulate phosphorylation of SIK2 (Fig. 9). However, glucagon can switch on the gluconeogenic gene programme through multiple distinct pathways (possibly involving both SIK-independent and -dependent pathways) whose precise mechanism is still under intensive investigation.

## Methods

### Materials

Forskolin, MK-2206 and PI-103 were from Tocris (Avon, UK). Protein G-Sepharose, glutathione-Sepharose and enhanced chemiluminescence reagents were purchased from GE Healthcare (Piscataway, NJ, USA). [γ^32^P]-labelled ATP was from Perkin Elmer (MA, USA). Precision Plus protein markers and SsoFast EvaGreen Supermix were from Bio-Rad (Hertfordshire, UK). Insulin-like growth factor 1 (IGF-1) was from Cell Signaling Technology (Hertfordshire, UK). Protease inhibitor cocktail tablets were purchased from Roche (Lewes, UK). Anti-HA-agarose, anti-FLAG-agarose, collagenase (from clostridium histolyticum type IV, Cat no. C5138), triiodothyronine, Bt_2_-cAMP and lactate were from Sigma-Aldrich (Poole, UK). Infinity glucose assay kit was from Thermo Scientific (Essex, UK). Insulin and glucagon were from Novo Nordisk (Sussex, UK). Cell culture media and reagents including Dulbecco’s modified eagle medium (DMEM) and M199 with glutamax were from Life Technologies (Paisley, UK). All other chemicals unless specified were obtained from Sigma-Aldrich.

### DNA constructs

Human *SIK1* (NM_173354), human *SIK2* (XM_041314) and human *SIK3* sequences (XM_005271481.1) were cloned into pCMV5 vector using standard molecular biology techniques. Site-directed mutagenesis technique was used for generating all of the mutants that were used in this manuscript. AdEasy expression system (Agilent technologies, Cheshire, UK) was used to generate recombinant adenovirus for expression of the WT SIK2, SIK2 phosphorylation-site mutants (Ser/Thr to Ala) and SIK2 drug-resistant mutant (T96Q) under the regulation of the pCMV promoter. Recombinant adenoviruses were generated and titered according to the manufacturer’s instructions.

### Antibodies

SIK2 (#6919), pSer473 Akt (#9271), pThr308 Akt (#9275), Akt2 (#2964), pSer133 CREB (#9198), CREB (#9197), p-Akt substrate (#9614), p-PKA substrate (#9621), pSer157 VASP (#3111), pSer246 HDAC4 (#3443), IP3R (#1178), pSer1756 IP3 receptor (#3760), AMPKα (#2532), pThr172 AMPK (#2535), ACC (#3662), pSer79 ACC (#3661) and PEPCK (#6924) antibodies were obtained from Cell Signaling Technology. CRTC2 (ST1099) and Tubulin (CP06) antibodies were purchased from Calbiochem (Nottingham, UK). CRTC3 (Ab91654) and GAPDH (Ab8245) antibodies were obtained from Abcam (Cambridge, UK), while anti-HA (12013819001) antibody was purchased from Roche. Secondary antibodies were obtained from Jackson ImmunoResearch (Suffolk, UK). Antibodies against SIK1, SIK2 and SIK3 (ref. 12), pThr182 SIK1 (recognizes equivalent T-loop site of SIK2/3; ref. 28) and pSer358 SIK2 (ref. 17) have been described previously. Antibodies against full-length human VASP (S407B, first bleed) and GFP (residues 2–238, S268B, first bleed) were generated in-house. Antibodies against pSer343 SIK2 (LKSHRSpSFPVEQR, residues 337–349 of human SIK2) and pSer484 SIK2 (GQRRHpTLSEVTNQ, residues 479–491 of human SIK2) were generated by YenZym custom antibody production service (South San Francisco, CA, USA). pSer577 SIK1 (recognizes equivalent pSer587 SIK2) was a gift from Dr Hiroshi Takemori (National Institute of Biomedical Innovation, Osaka, Japan) and has been described previously^29^. pSer33 PFKFB1 antibody has been described previously^10^.

### Protein sequence

Protein sequences for SIK1 (*Homo sapiens*, P57059), SIK2 (*Homo sapiens*, Q9H0K1) and CRTC2 (*Homo sapiens*, Q53ET0) were obtained from the Swiss-Prot database while SIK3 sequence was obtained from NCBI (XP_005271538.1).

### Animals

Animal studies were approved by the University of Dundee and the Paris Descartes University ethics committee (no. CEEA34.BV.157.12) and performed under a UK Home Office project licence or a French authorization to experiment on vertebrates (no.75–886) in accordance with the European guidelines or the University of Pennsylvania IACUC in accordance with the National Institute of Health guidelines. Generation of the Akt2^−/−^ mice has been described previously^18^. Liver-specific AMPKα1/α2 KO mice have been described previously^4^.

SIK2 (*Snf1lk2*) targeting construct was generated from PCR products amplified from the DNA of 129/Sv ES cells by the *pfx* polymerase (Invitrogen). The 5′ and 3′ homology arms, both 3.4 kb in length, were inserted on either side of a PGK promoter-driven hygromycin selection cassette flanked by FLP Recognition Target (FRT) sites, into the pL3-FRT-Hygro vector. A 1.1-kb fragment of genomic DNA bearing exon 5, encoding partially the SIK2 catalytic domain including the phosphorylation-site Thr175 within the activation loop (corresponding to amino acids 160–201), flanked by loxP sites, was introduced between the hygromycin resistance cassette and the 3′ homology arm (Fig. 2a). Exponentially growing 129/SV CK35 embryonic stem cells^30^ were electroporated with the linearized target construct DNA and selected on plates containing hygromycin. The targeted clones were identified by PCR across both homology arms, with confirmation by Southern blot analysis. Cell populations expanded from the targeted clones were injected into C57BL/6 eight-cell embryos with a laser-assisted micromanipulation system, and animals displaying germline transmission were mated with C57BL/6J mice. The hygromycin resistance cassette flanked by FRT sites were excised by crossing SIK2^lox/+^ mice with flippase (FLP)-expressing mice^31^. The resulting heterozygous offspring were backcrossed to at least four generations into the C57BL/6J background. Liver disruption of exon 5 flanked by loxP sites was achieved by crossing SIK2-floxed mice with Alfp–Cre transgenic mice^32^ to generate SIK2^lox/lox^ (control) and SIK2^lox/lox^–Alfp–Cre (liver KO) mice. Routine genotyping was carried out by multiplex PCR on tail DNA with the P1 (5′-gtagtttacattagcacattggtgcctc-3′), P2 (5′-cctagaatgcactctgcaaacactggacac-3′) and P3 (5′-tctacatggagggtgtcgcagagctccatg-3′) primers, to yield amplification products of 391 bp (WT allele) and 487 bp (floxed allele) with P1/P2 and 708 bp (KO allele) with P1/P3. Liver TKO of AMPKα1, AMPKα2 and SIK2 was achieved by crossing SIK2^lox/lox^–Alfp–Cre mice with AMPKα1^lox/lox^, α2^lox/lox^ mice to generate AMPKα1^lox/lox^, α2^lox/lox^, SIK2^lox/lox^ (control) and AMPKα1^lox/lox^, α2^lox/lox^, SIK2^lox/lox^–Alfp–Cre (liver TKO) mice. Ten-week-old male mice were used in all experiments.

Liver-specific LKB1 KO mice were generated by crossing LKB1^lox/lox^ mice (kindly provided by Ronald DePinho, Harvard University)^33^ with tamoxifen-inducible albumin–Cre–ERT2 mice (kindly provided by Daniel Metzger, IGBMC, France)^34^. Ten-week-old male LKB1^lox/lox^–Alb–Cre–ERT2 mice were treated either with vehicle containing sunflower oil and ethanol 10% (control) or tamoxifen (Sigma) at 1 mg per mouse injected intraperitoneally in a final volume of 100 μl for 5 consecutive days. Mice were studied or used for primary hepatocyte isolation 3 weeks after the start of tamoxifen administration. Male C57BL/6 mice were obtained from Harlan (UK) and 8–12-week-old male mice were used for experiments. All mice were maintained on a standard chow diet (or high-carbohydrate diet described in Supplementary section) and 12/12 h light/dark in a barrier facility.

### Fasting/refeeding and oral glucose tolerance test

For the fasting–refeeding experiment, mice were either fasted for 18 h or fasted for 24 h then refed a high-carbohydrate diet (Harlan TD.08247) for 12 h. Oral glucose tolerance test was performed on mice fasted for 16 h as previously described^4^. Blood glucose levels were determined at 0, 20, 40, 60, 90 and 120 min after oral administration of glucose (3 g kg^−1^ body weight) using a glucometer (Roche Diagnostics).

### Primary hepatocyte culture and glucose production measurement

Primary hepatocytes were isolated from fed 10-week-old male mice by a modified version of the collagenase method^35^ Hepatocyte preparations that displayed a cell viability ≥90% were used for experiments. Isolated hepatocytes were plated in 12-well plates at a density of 1.25 × 10^5^ per well in the plating medium (M199 with glutamax supplemented with 100 U ml^−1^ penicillin, 0.1 mg ml^−1^ streptomycin, 0.1% bovine serum albumin (BSA), 10% (v/v) foetal bovine serum, 10 nM insulin, 200 nM triiodothyronine, 500 nM dexamethasone) for 4–6 h and then incubated with overnight medium (M199 with glutamax supplemented with 100 U ml^−1^ penicillin, 0.1 mg ml^−1^ streptomycin and 100 nM dexamethasone). The next morning, cells were washed once with 1 ml of PBS and 750 μl of the assay medium (containing DMEM without glucose, 100 U ml^−1^ penicillin and 0.1 mg ml^−1^ streptomycin, 100 nM dexamethasone, 1 mM sodium pyruvate and 10 mM lactate, pH 7.4) was added per well. The cells were stimulated with the respective compounds or hormones for the indicated time described in the figure legends and glucose production (glucose in the medium) was measured using the hexokinase/glucose-6-phosphate dehydrogenase method^4^,^36^.

### Preparation and infection of adenovirus

Generation of recombinant adenovirus carrying genes of our interest (GFP and GFP- or HA-tagged SIK2 wild type and mutants) was carried out either using AdEasy system (Agilent Technologies, Berkshire, UK) according to manufacturer’s protocol and the adenovirus was purified using caesium chloride banding according to the protocol described by Luo *et al.*^37^ in house or generated and purified by Vector Biolabs (CA, USA). Adenoviruses were added to the cells at a defined multiplicity of infection (MOI). One MOI equals one infectious viral particle per cell. For overexpression studies in primary hepatocytes, the cells were infected with the indicated MOI for 16 h prior to cell lysis.

### Immunoprecipitation of HA–SIK2 for mass spectrometry

Primary mouse hepatocytes were infected with adenoviruses encoding WT or mutant SIK2 at 1:25 MOI for 16 h. These cells were left untreated or stimulated with 10 nM insulin or 100 nM glucagon for 10 min and lysed. HA–SIK2 was immunoprecipitated from 5 mg of clarified lysates with 30 μl of anti-HA-agarose beads. The washed beads were heated with 1 × NuPAGE LDS sample buffer (Invitrogen) and 10 mM DTT for 5 min at 70 °C. Samples were further incubated with 50 mM iodoacetamide for 30 min to alkylate cysteine residues and electrophoresed on NuPAGE 4–12% Bis-Tris gels (Invitrogen). The gels were colloidal coomassie stained and HA–SIK2 bands were excised and processed as below.

### Sample preparation for mass spectrometry

Samples were prepared in a laminar flow hood to reduce contamination. The gel pieces were cut and washed for 10 min with 500 μl of water, 50% acetonitrile/water, 0.1 M ammonium bicarbonate (NH_4_HCO_3_) and 50% acetonitrile/50 mM NH_4_HCO_3_. Washes were carried out on a vibrax-shaking platform and all liquid was removed between washes. The final step was repeated until the gel pieces were colourless. Acetonitrile (0.3 ml) was added to these gel pieces and removed after 15 min. The gel pieces were dried in a centrifugal-evaporator (Speed-Vac). The protein in the dried gel pieces was digested in 30 μl of 25 mM triethylammonium bicarbonate containing 5 μg ml^−1^ of trypsin at 30 °C overnight on a shaking platform. Subsequently, an equivalent volume of acetonitrile was added for 15 min. The supernatant was collected in a clean tube and dried using a Speed-Vac. Meanwhile 100 μl of 50% acetonitrile/2.5% formic acid was added to the gel pieces and incubated for 15 min. The supernatant was combined with the first dried extract and dried in a Speed-Vac and stored at −20 °C.

### Phosphorylation-site identification by mass spectrometry

Phospho-peptides and their approximate relative abundances were initially identified by LC-MS-MS mass spectrometry on an Applied Biosystems 4000 QTrap coupled to a Dionex Ultimate 3000 liquid chromatography system. The MS was set up to use a precursor ion scan of m/z 79 in negative ion mode followed by an ion trap high resolution and a high sensitivity MS-MS scan in positive mode^38^. QTrap precursor ion analysis was used to allow simple observation of variations in phosphopeptide intensity from one treatment to another. Data was analysesd using Analyst software (ABSciex). Phospho-peptides were also further analysed by LC-MS-MS on a Thermo LTQ-Orbitrap Classic equipped with a nanoelectrospray ion source (Proxeon Biosystems) and coupled to a Proxeon EASY-nLC system. Multi-Stage-Activation was used to provide an MS3 scan of any parent ions showing a neutral loss of 48.9885, 32.6570, 24.4942, allowing for 2+, 3+ and 4+ charge states, respectively. The resulting MS3 scan was automatically combined with the relevant MS2 scan prior to data analysis. Results were searched against an in-house database containing the SIK sequences using the Mascot algorithm ( www.matrixscience.com) to identify peptides. Phosphorylation sites on the peptides were also analyzed with Xcalibur software and Proteome Discoverer (Thermo Scientific).

### Quantitative PCR analysis

Total RNA from primary hepatocytes and mouse liver tissue was extracted using Trizol (Invitrogen), and single-strand complementary DNA was synthesized from 5 μg of total RNA with random hexamer primers (Applied Biosystems) and Superscript II (Invitrogen). Real-time reverse transcription–PCRs were carried out in a final volume of 20 μl containing 125 ng of reverse-transcribed total RNA, 500 nM of primers and 10 μl of 2 × PCR mix containing Sybr Green (Roche). The reactions were performed in 96-well plates in a LightCycler 480 instrument (Roche) with 40 cycles. We determined the relative amounts of the mRNAs studied by means of the second-derivative maximum method, with LightCycler 480 analysis software and 18S RNA as the invariant control for all studies. The sense and antisense PCR primers used, respectively, were as follows: for *Ppargc-1*α, 5′-ATACCGCAAAGAGCACGAGAAG-3′, 5′-CTCAAGAGCAGCGAAAGCGTCACAG-3′; for *Pck1*, 5′-GTGCTGGAGTGGATGTTCGG-3′, 5′-CTGGCTGATTCTCTGTTTCAGG-3′; for *G6pc*, 5′-ACTGTGGGCATCAATCTCCTC-3′, 5′-CGGGACAGACAGACGTTCAGC-3′; for *18S*, 5′-GTAACCCGTTGAACCCCATT-3′, 5′-CCATCCAATCGGTAGTAGCG-3′; for *Fasn*, 5′-AGCGGCCATTTCCATTGCCC-3′, 5′-CCATGCCCAGAGGGTGGTTG-3′; for *Srebp-1c*, 5′-GGAGCCATGGATTGCACATT-3′, 5′-GCTTCCAGAGAGGAGGCCAG-3′.

### General cell culture

HEK293 and Ad-293 cells were cultured in DMEM supplemented with 10% (v/v) foetal bovine serum, 2 mM L-glutamine, 100 U ml^−1^ penicillin and 0.1 mg ml^−1^ streptomycin. They were maintained in T75 flasks at 50–80% confluence for a maximum of 20 passages. Cells were split at 70–80% confluence and plated in a new dish or flask after an appropriate dilution. Cells were serum starved overnight before stimulating with hormones, growth factors or kinase inhibitors.

### Cell lysis

Medium was aspirated and cells were rinsed once with ice-cold PBS. Cells were subsequently lysed on ice using ice-cold lysis buffer (50 mM Tris-HCl, pH 7.5, 1 mM EGTA, 1 mM EDTA, 1% (w/v) Triton X-100, 1 mM Na_3_VO_4_, 50 mM NaF, 5 mM sodium pyrophosphate, 0.27 M sucrose, 0.1% (v/v) 2-mercaptoethanol and ‘complete’ protease inhibitor cocktail). Whole-cell lysate was cleared of insoluble particles by centrifugation at 4 °C, 13,000*g* for 10 min. The supernatant was removed and stored at −80 °C.

### Preparation of tissue lysates

Frozen liver samples were homogenized using a rotor-stator homogenizer (Polytron, Kinematica AG) in 20 volumes of ice-cold lysis buffer, clarified for 10 min at 13,000*g* at 4 °C and protein concentration determined using Bradford reagent (Pierce) with BSA as standard. Lysates were stored at −80 °C.

### Immunoprecipitation of endogenous SIK2 protein

SIK2 antibody or pre-immune immunoglobulin G (2–3 μg; as a control) was incubated with 5 μl of protein G-Sepharose for 1 h at 4 °C on an orbital shaker. This mixture was then added to 0.5–2 mg of cell/tissue extracts and further incubated at 4 °C for 1 h prior to isolation by centrifugation. The resultant precipitates were washed and used for immunoblotting or kinase assays as follows (and also shown previously^17^,^39^).

### Immunobloting

Cell or tissue lysates (20–40 μg) were denatured in Laemmli buffer, separated by SDS–polyacrylamide gel electrophoresis and transferred to nitrocellulose. Membranes were blocked in 50 mM Tris-HCl pH 7.6, 137 mM NaCl and 0.1% (v/v) Tween-20 containing 10% (w/v) skimmed milk or 5% (w/v) BSA for 1 h at room temperature and incubated overnight at 4 °C with the indicated primary antibodies. Detection was performed using horseradish peroxidase-conjugated secondary antibodies and enhanced chemiluminescence reagent. Uncropped images of immunoblots are shown in Supplementary Fig. 12.

### SIK activity assays

SIK isoforms were immunoprecipitated from liver tissue/hepatocyte extracts (0.5–2 mg). SIK activity assay was performed in a final volume of 50 μl containing 15 μl of protein kinase (immune purified/precipitated) and 35 μl of reaction mix. The reaction mix contained 200 μM of Sakamoto-tide peptide (ALNRTSSDSALHRRR), 50 mM HEPES, 1 mM EGTA, 10 mM magnesium acetate and 0.1 mM [γ^32^P]-labelled ATP. Control reactions either lacked purified kinase or contained pre-immune immunoglobulin G-coupled beads. Phosphorylation reactions were initiated by adding reaction mix to the protein kinase and incubating on a thermomixer at 30 °C for 20 min. These reactions were terminated by spotting 40 μl of mixture onto P81 phosphocellulose paper and immersing them in 75 mM orthophosphoric acid. P81 papers were washed three times for 10 min with 75 mM orthophosphoric acid and finally in acetone for 5 min. P81 papers were air-dried and the incorporation of ^32^P into peptide substrate was determined by Cherenkov counting. Results were expressed in mU mg^−1^ protein where 1 mU equals 1 pmol of phosphate incorporation into a substrate in 1 min at 30 °C (refs 17, 39).

### Immunofluorescence

Primary mouse hepatocytes were fixed on glass cover slips with 3.7% paraformaldehyde after the treatment indicated in figure legends. Cells were washed three times with PBS containing 5% (w/v) BSA followed by incubation with 0.1% NP-40 in PBS for 5 min. Subsequently, cells were blocked with PBS containing 5% (w/v) BSA for 20 min and incubated with anti-rabbit SIK2 antibody (Cell Signaling, #6924) or anti-rabbit CRTC2 antibody (Calbiochem, Cat no. ST1099) at a dilution of 1:500 for 1 h at 37 °C. Excess antibodies were removed by washing the cells three times with PBS containing 5% (w/v) BSA. This was followed by a 30 min incubation with secondary antibody (Alexa Fluor 488). Subsequently the cells were further washed three times with PBS containing 5% (w/v) BSA before mounting the cover slips on a glass slide with mounting media containing 4′,6-diamidino-2-phenylindole. Cells were later analyzed with a Nikon Eclipse Ti fluorescent microscope and images were captured using Nikon NIS Elements BR 3.1 software.

### Statistics

Data are expressed as mean±standard deviation or standard error. Statistical analysis was performed using Student’s *t*-test or one-way or two-way analysis of variance using Tukey’s or Dunnet’s test for multiple comparisons where appropriate. Differences between groups were considered statistically significant at *P*<0.05.

## Author contributions

K.S. and M.F. came up with the concept of the study. K.S., M.F., C.S. and K.P. designed the overall experiments. K.P. performed the experiments and analysis shown in Figs 1, 6, 7 and 8 (and the associated data shown in Supplementary Section). K.S. and C.S. supervised K.P. M.F. generated AMPKα1 floxed mice and SIK2-floxed mice, designed and performed the experiments shown in Figs 2, 3,4, 5 and 7 and interpreted data. A.M. and N.B. analyzed samples of experiments shown in Figs 2, 3,4, 5 and 7. M.F. supervised A.M. and N.B. E.T. handled ES cells for the generation of SIK2-floxed mouse. D.G.C. and R.G. performed mass spectrometry analysis and provided data interpretation shown in Fig. 1a,b (and Supplementary Figs 1 and 3). M.D. and M.P. performed molecular cloning. O.G. provided Ser358 SIK2 antibody, performed a part of SIK2 phospho-antibody characterization and edited the draft. K.H.K. provided CRTC2 floxed mice. P.T. performed experiments and analyzed data shown in Supplementary Fig. 11, and M.W. made a contribution to the experiment shown in Fig. 1d and Supplementary Fig. 11. B.V. provided AMPKα2 floxed mice and edited the draft. M.J.B. supervised P.T. and M.W. and edited the draft. N.G. provided HG-9-91-01 and KN112 compounds and edited the draft. K.S. mainly wrote the manuscript and M.F. and C.S. made key contributions to draft writing and other co-authors contributed and edited the draft.

## Additional information

**Accession codes:** The mass spectrometry data has been deposited to the ProteomeXchange Consortium^40^ via the PRIDE partner repository under the accession code PXD001032.

**How to cite this article**: Patel, K. *et al.* The LKB1-Salt-inducible kinase pathway functions as a key gluconeogenic suppressor in the liver. *Nat. Commun.* 5:4535 doi: 10.1038/ncomms5535 (2014).

## Supplementary Material

Supplementary Information Supplementary Figures 1-12
