# Supplementary material for: The LKB1-salt-inducible kinase pathway functions as a key gluconeogenic suppressor in the liver
Source: Nat Commun. 2014 Aug 4;5:4535. doi: 10.1038/ncomms5535 (PMC4143937; doi:10.1038/ncomms5535)
Supplement: Supplementary Information — Supplementary Figures 1-12 [file ncomms5535-s1.pdf]

A: [related to Fig 1B (Line1)]: OrbiTrap MS2 data for m/z 515.2 2^+ :[M+H] = 1029.44
SIK2(Glucagon treatment) SsFPVEQR S343

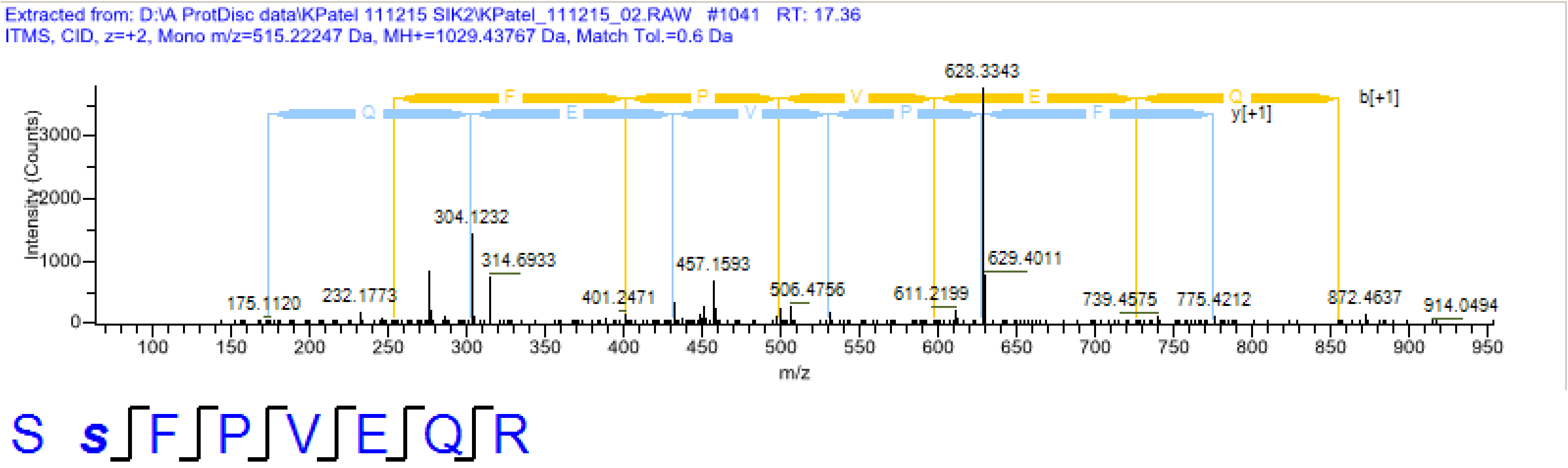

	(NH3+)-S	s/F	F/P	P/V	V/E	E/Q	Q/R	R-(COOH)
b (+1)	88.0	**255.0**	**402.1**	**499.2**	**598.2**	**727.2**	**855.3**	1011.4
y (+1)	942.4	**775.4**	**628.3**	**531.3**	**432.2**	**303.2**	**175.1**	1029.4

This OrbiTrap data is ambiguous as it does not define sS or Ss, whereas QTrap data from SIK2 (Glucagon treatment) analyzed in Mascot does, see below.

A: continued [related to Fig 1B (Line1)]: QTrap MS2 data for m/z 515.30 2+ :[M+H] = 1029.57
SIK2(Glucagon treatment) SsFPVEQR S343

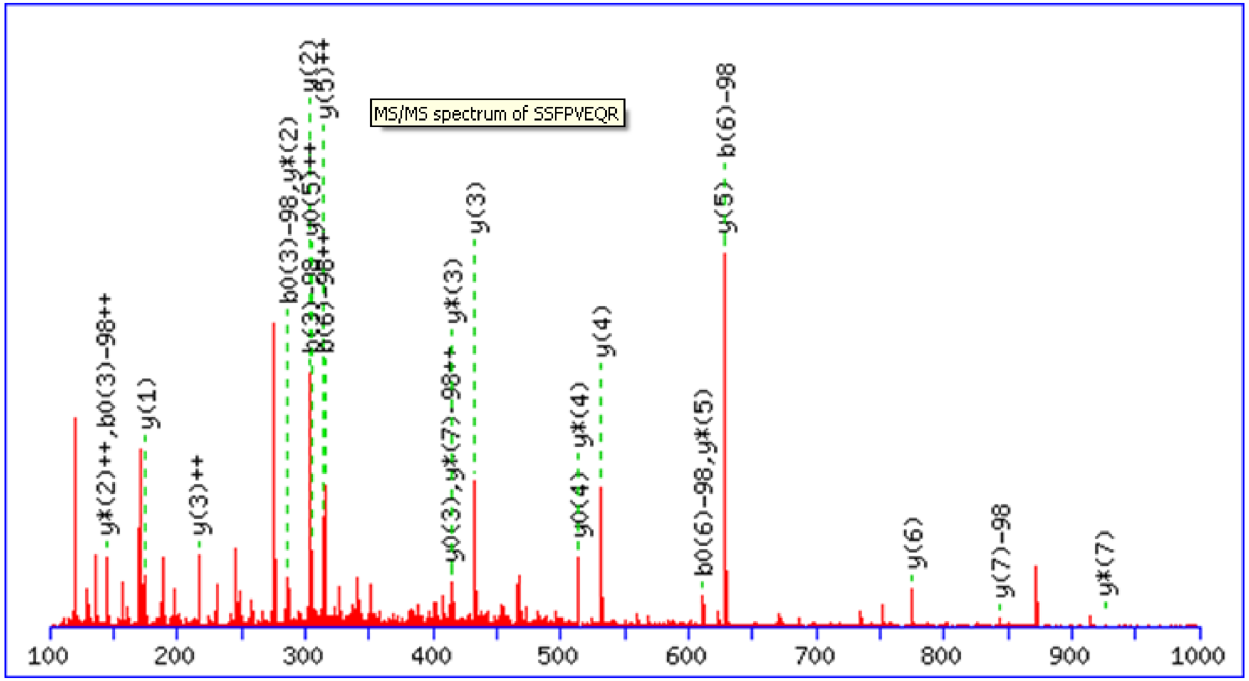

#	b	b^{++}	b^{*}	b^{*++}	b^{0}	b^{0++}	Seq.	y	y^{++}	y^{*}	y^{*++}	y^{0}	y^{0++}	#
1	88.0393	44.5233			70.0287	35.5180	S							8
2	157.0608	79.0340			139.0502	70.0287	S	844.4312	422.7192	827.4046	414.2060	826.4206	413.7139	7
3	304.1292	152.5682			286.1186	143.5629	F	775.4097	388.2085	758.3832	379.6952	757.3991	379.2032	6
4	401.1819	201.0946			383.1714	192.0893	P	628.3413	314.6743	611.3148	306.1610	610.3307	305.6690	5
5	500.2504	250.6288			482.2398	241.6235	V	531.2885	266.1479	514.2620	257.6346	513.2780	257.1426	4
6	629.2930	315.1501			611.2824	306.1448	E	432.2201	216.6137	415.1936	208.1004	414.2096	207.6084	3
7	757.3515	379.1794	740.3250	370.6661	739.3410	370.1741	Q	303.1775	152.0924	286.1510	143.5791			2
8							R	175.1190	88.0631	158.0924	79.5498			1

B: [related to Fig 1B (Line2)]: OrbiTrap MS2 data for m/z 690.8 2^+ :[M+H] = 1380.69

SIK2 (Glucagon treatment) RPsTIAEQTVAK <u>**S358**</u>

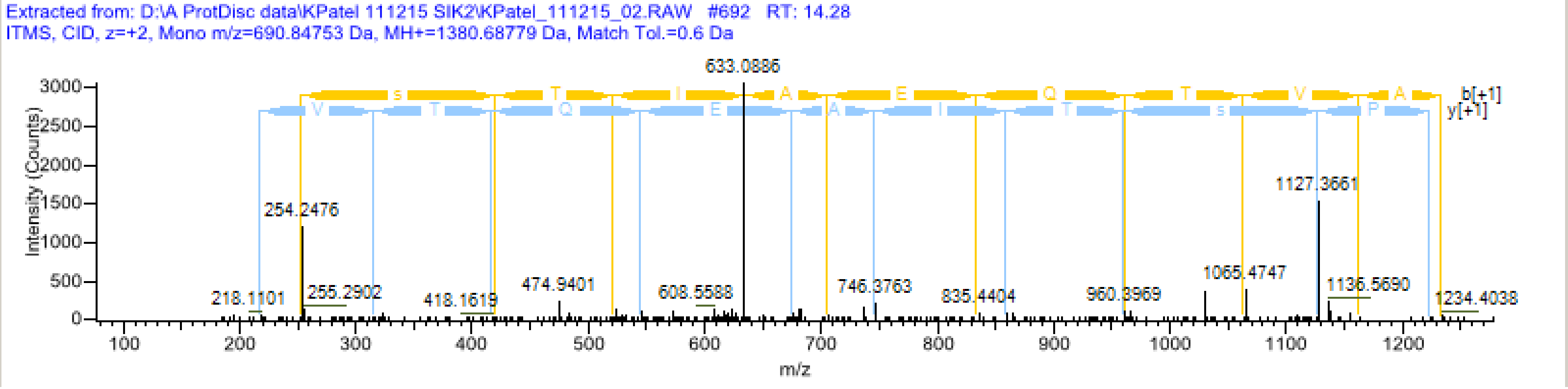

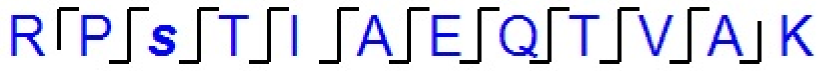

	(NH3+)-R	P/s	s/T	T/I	I/A	A/E	E/Q	Q/T	T/V
b (+1)	157.1	**254.2**	**421.2**	**522.2**	**635.3**	**706.3**	**835.4**	**963.4**	**1064.5**
y (+1)	**1224.6**	**1127.5**	**960.5**	**859.5**	**746.4**	**675.4**	**546.3**	**418.3**	**317.2**

	V/A	A/K	K-(COOH)
b (+1)	**1163.5**	**1234.6**	1362.7
y (+1)	**218.1**	147.1	1380.7

C: [related to Fig 1B (Line3)]: OrbiTrap MS2 data for m/z 909.9 2^+ :[M+H] = 1818.84
SIK2 (Glucagon treatment) AQTVGLPVTMHsPNMR S379

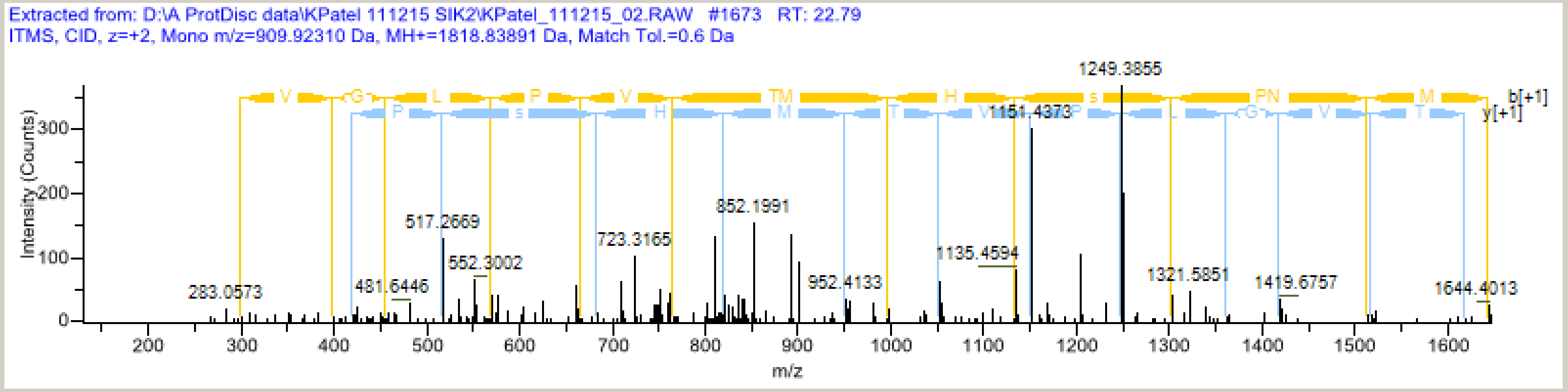

A Q T V G L P V T M H **s** P N M R

	(NH3+)-A	Q/T	T/V	V/G	G/L	L/P	P/V	V/T	T/M
b (+1)	72.0	200.1	**301.2**	**400.2**	**457.2**	**570.3**	**667.4**	**766.4**	867.5
y (+1)	1747.8	1619.7	**1518.7**	**1419.6**	**1362.6**	**1249.5**	**1152.5**	**1053.4**	**952.4**

	M/H	H/s	s/P	P/N	N/M	M/R	R-(COOH)
b (+1)	**998.5**	**1135.6**	**1302.6**	1399.6	**1513.7**	**1644.7**	1800.8
y (+1)	**821.3**	**684.3**	**517.3**	**420.2**	306.2	175.1	1818.8

D: [related to Fig 1B (Line4)]: OrbiTrap MS2 data for m/z 1059.0 2^{+} :[M+H] = 2117.05
SIK2 (Glucagon treatment) RHtLSEVTNQLVVMPGAGK <u>**T484**</u>

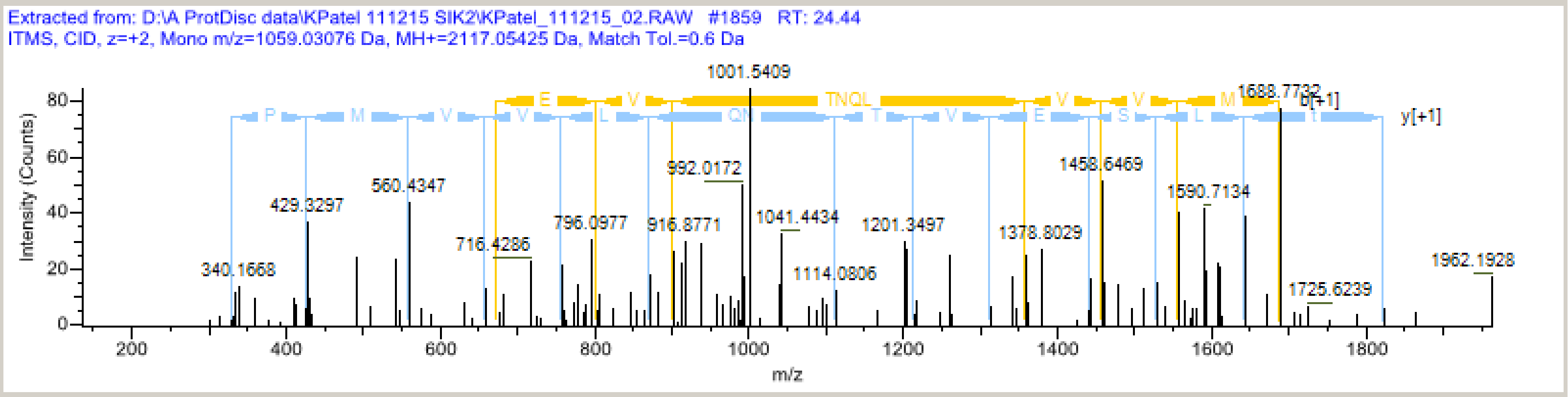

R H t L S E V T N Q L V V M P G A G K

	(NH3+)-R	H/t	t/L	L/S	S/E	E/V	V/T	T/N	N/Q
b (+1)	157.1	294.2	475.2	588.3	**675.3**	**804.3**	**903.4**	1004.5	1118.5
y (+1)	1961.0	**1823.97**	**1642.9**	**1529.8**	**1442.8**	**1313.76**	**1214.7**	**1113.6**	999.6

	Q/L	L/V	V/V	V/M	M/P	P/G	G/A	A/G	G/K
b (+1)	1246.6	**1359.6**	**1458.7**	**1557.8**	**1688.8**	1785.9	1842.9	1913.9	1971.0
y (+1)	**871.5**	**758.4**	**659.4**	**560.3**	**429.2**	**332.2**	275.2	204.1	147.1

	K-(COOH)
b (+1)	2099.0
y (+1)	2117.1

E: [related to Fig 1B (Line4)]: OrbiTrap MS2 data for m/z 711.7 3^+ :[M+H] = 2133.05
SIK2 (Glucagon treatment) RHTLsEVTNQLVVmPGAGK S486

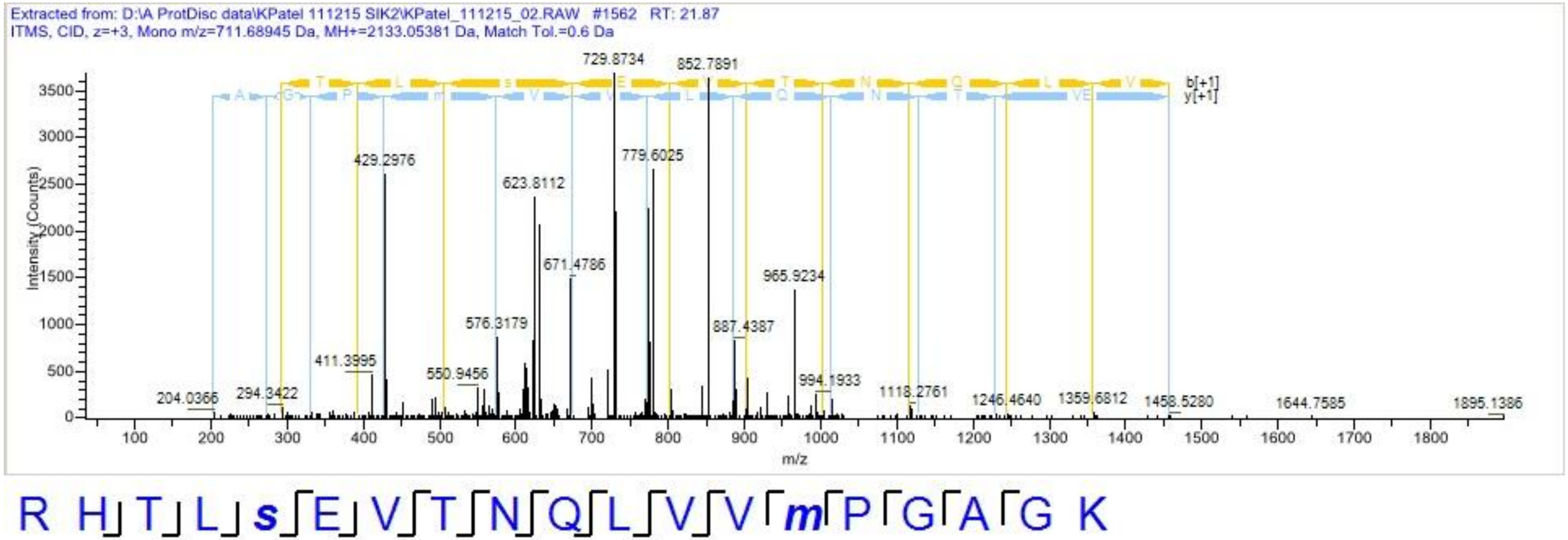

	(NH3+)-R	H/T	T/L	L/s	s/E	E/V	V/T	T/N	N/Q
b (+1)	157.1	**294.1**	**395.2**	**508.3**	**675.3**	**804.3**	**903.4**	**1004.5**	**1118.5**
y (+1)	1977.0	1839.9	1738.8	1625.8	**1458.8**	1329.7	**1230.7**	**1129.6**	**1015.6**

	Q/L	L/V	V/V	V/M	M/P	P/G	G/A	A/G	G/K
b (+1)	**1246.6**	**1359.6**	1458.7	1557.8	1704.8	1801.9	1858.9	1929.9	1986.9
y (+1)	**887.5**	**774.4**	**675.3**	**576.3**	**429.2**	**332.2**	**275.2**	**204.1**	147.1

	K-(COOH)
b (+1)	2115.0

F: [related to Fig 1B (Line5)]: OrbiTrap MS2 data for m/z 851.4 2$^+$:[M+H] = 1701.83
SIK2 (Glucagon treatment) RAsDTSLTQGIVAFR **S587**

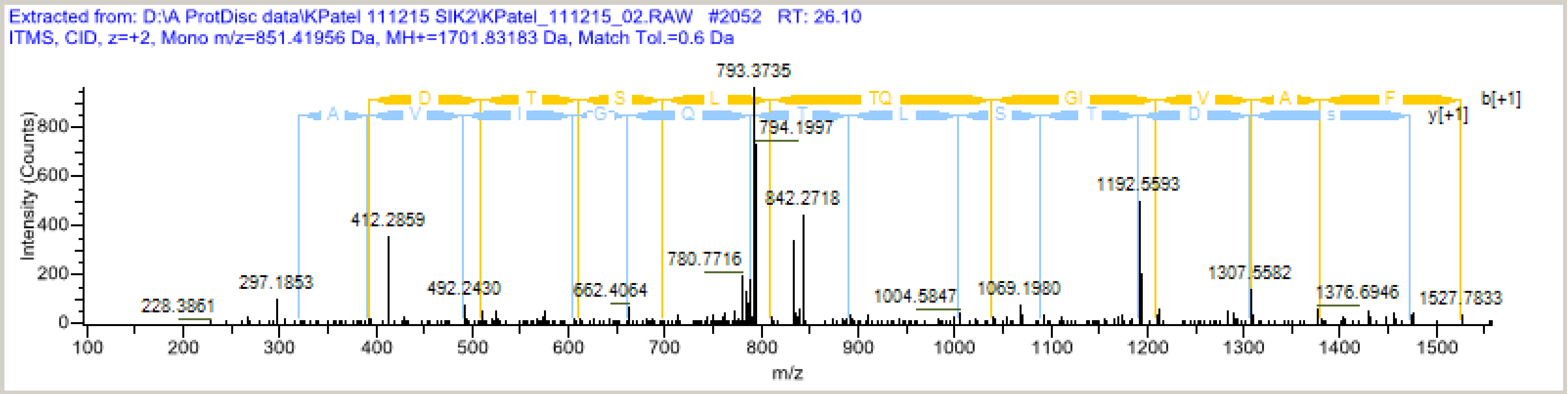

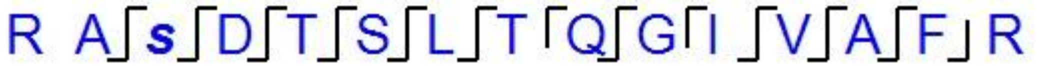

	(NH3+)-R	A/s	s/D	D/T	T/S	S/L	L/T	T/Q	Q/G
b (+1)	157.1	**228.14**	**395.1**	**510.2**	**611.2**	**698.3**	**811.3**	912.4	**1040.4**
y (+1)	**1545.7**	**1474.7**	**1307.7**	**1192.7**	**1091.6**	**1004.6**	**891.5**	**790.5**	**662.4**

	G/I	I/V	V/A	A/F	F/R	R-(COOH)
b (+1)	1097.5	**1210.5**	**1309.6**	**1380.7**	**1527.7**	1683.8
y (+1)	**605.4**	**492.3**	**393.2**	**322.2**	175.1	1701.8

Supplementary Figure 1: Annotated MS2 spectra for SIK2 phosphopeptides listed in Figure 1B.
Figures of the spectra from Orbitrap LC-MS-MS analyses were derived from Proteome Discoverer software (Thermo) and that from QTrap LC-MS-MS analysis was derived from Mascot software (www.matrixscience.com). Ions marked in bold in the tables are those detected in the spectra. Phosphorylation sites are indicated by lower case (e.g. s, t).

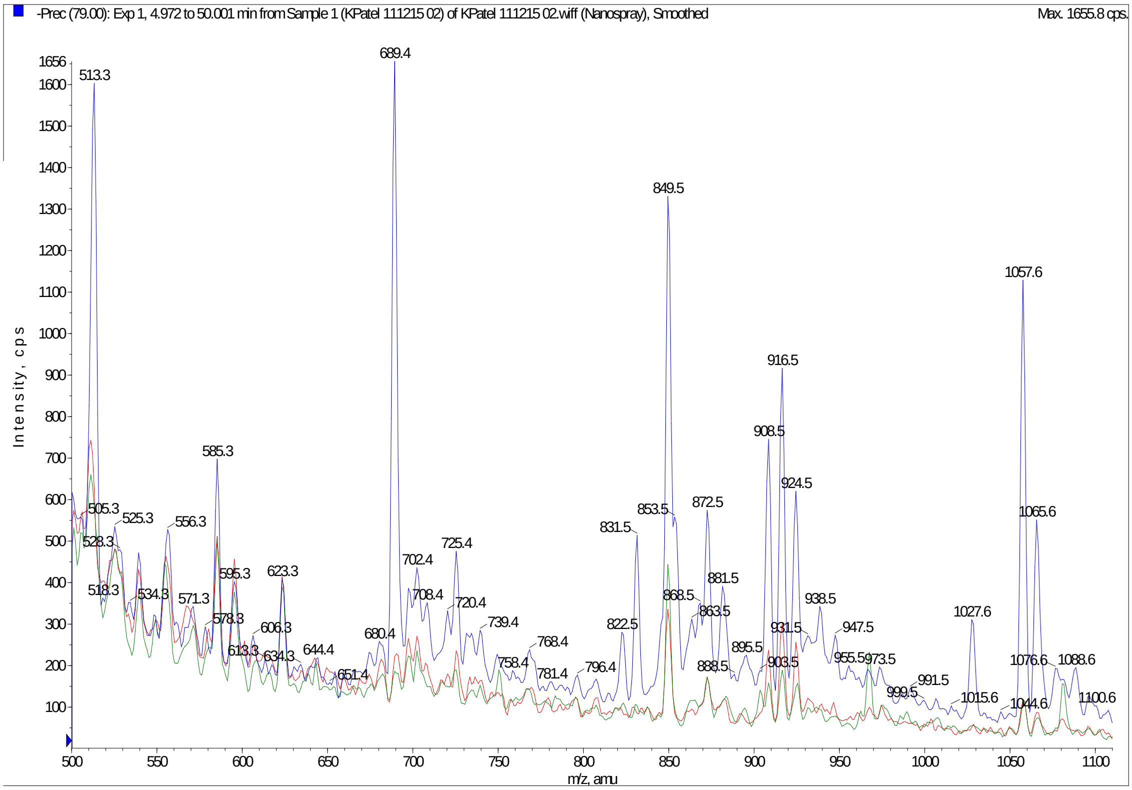

Supplementary Figure 2: Precursor ion scanning (-79Da) of SIK2 tryptic digests

SIK2 immunoprecipitated samples from control (Green), glucagon-treated (Blue) and insulin-treated (Red) cells were trypsin-digested and analyzed by precursor ion scanning (-79Da). Phosphopeptides and their approximate relative abundances were identified by LC-MS-MS mass spectrometry on an Applied Biosystems 4000 QTrap coupled to a Dionex Ultimate 3000 liquid chromatography system. The MS was set up to use a precursor ion scan of m/z -79 in negative ion mode followed by an ion trap high resolution and a high sensitivity MS-MS scan in positive mode. Intensity of (-79) signal is plotted against m/z (for negative mode Average mass). Peaks that increase on glucagon treatment are 513.3 (SsFPVEQR, S343); 689.4 (RPsTIAEQTVAK, S358); 849.5 (RAsDTSLTQGIVAFR, S587); 908.5, 916.5, 924.5 (AQTVGLPVTMHsPNMR, S379, where the triplet of peaks is caused by methionine oxidation) and 1057.6 (RHtLSEVTNQLVVMPGAGK, T484).

#1

1 MVMADGPRHL QRGPVR**VGFY DIEGTLGKGN FAVVK**LGRHR ITKTEVAIK**I**
51 **IDKSQLDAVN LEK**IYR**EVQI MKMLDHPHII KLYQVMETKS MLYLVTEYAK**
101 **NGEIFDYLAN HGRLNESEAR RKFWQILSAV DYCHGR**KIVH R**DLKAENLLL**
151 **DNNMNIKIAD FGFGNFFKSG ELLATWCGSP PYAAPEVFEG QQYEGPQLDI**
201 **WSMGVVLYVL VCGALPFDGP TLPILR**QRVL EGR**FRIPYFM SEDCEHLIRR**
251 **MLVLDPSKRL TIAQIK**EHK**W MLIEVPVQRP VLYPQEQENE PSIGEFNEQV**
301 **LRLMHSLGID QQKTIESLQN KSYNHFAAIY FLLVER**LKSH R**SSFPVEQR**L
351 DGR**QRRPSTI AEQTVAKAQT VGLPVTMHSP NMR**LLR**SALL PQASNVEAFS**
401 **FPASGCQAEA AFMEEECVDT PKVNGCLLDP VPPVLVRKGC QSLPSNMMET**
451 **SIDEGLETEG EAEEDPAHAF EAFQSTR**SGQ R**RHTLSEVTN QLVVMPGAGK**
501 **IFSMNDSPSL DSVDSEYDMG SVQRDLNFLE DNPSLKDIML ANQPSPRMTS**
551 **PFISLRPTNP AMQALSSQK**R EVHNR**SPVSF R**EGR**RASDTS LTQGIVAFRQ**
601 **HLQNLAR**TK**G ILELNKVQLL YEQIGPEADP NLAPAAPQLQ DLASSCPQEE**
651 **VSQQQESVST LPASVHPQLS PRQSLETQYL QHR**LQKPSLL SK**AQNTCQLY**
701 **CKEPPRSLEQ QLQEHR**LQQK RLFLQK**QSQL QAYFNQMQIA ESSYPQPSQQ**
751 **LPLPR**QETPP PSQQAPPFSL TQPLSPVLEP SSEQMQYSPF LSQYQEMQLQ
801 PLPSTSGPR**A APPLPTQLQQ QQPPPPPPPP PPRQPGAAPA PLQFSYQTCE**
851 **LPSAASPAPD YPTPCQYPVD GAQQSDLTGP DCPR**SPGLQE APSSYDPLAL
901 SELPGLFDCE MLDAVDPQHN GYVLVN

#2

1 MVMADGPRHL QRGPVR**VGFY DIEGTLGKGN FAVVK**LGRHR ITKTEVAIK**I**
51 **IDKSQLDAVN LEK**IYR**EVQI MKMLDHPHII KLYQVMETKS MLYLVTEYAK**
101 **NGEIFDYLAN HGRLNESEAR RKFWQILSAV DYCHGR**KIVH R**DLKAENLLL**
151 **DNNMNIKIAD FGFGNFFK**SG ELLATWCGSP PYAAPEVFEG QQYEGPQLDI
201 WSMGVVLYVL VCGALPFDGP TLPILRQRVL EGR**FRIPYFM SEDCEHLIR**R
251 **MLVLDPSKRL TIAQIK**EHK**W MLIEVPVQRP VLYPQEQENE PSIGEFNEQV**
301 **LRLMHSLGID QQKTIESLQN KSYNHFAAIY FLLVER**LKSH R**SSFPVEQR**L
351 DGR**QRRPSTI AEQTVAKAQT VGLPVTMHSP NMR**LLR**SALL PQASNVEAFS**
401 **FPASGCQAEA AFMEEECVDT PKVNGCLLDP VPPVLVRKGC QSLPSNMMET**
451 **SIDEGLETEG EAEEDPAHAF EAFQSTR**SGQ R**RHTLSEVTN QLVVMPGAGK**
501 **IFSMNDSPSL DSVDSEYDMG SVQRDLNFLE DNPSLKDIML ANQPSPRMTS**
551 **PFISLRPTNP AMQALSSQK**R EVHNR**SPVSF R**EGR**RASDTS LTQGIVAFRQ**
601 **HLQNLAR**TK**G ILELNKVQLL YEQIGPEADP NLAPAAPQLQ DLASSCPQEE**
651 **VSQQQESVST LPASVHPQLS PRQSLETQYL QHRLQKPSLL SKAQNTCQLY**
701 **CK**EPPR**SLEQ QLQEHR**LQQK RLFLQK**QSQL QAYFNQMQIA ESSYPQPSQQ**
751 **LPLPRQETPP PSQQAPPFSL TQPLSPVLEP SSEQMQYSPF LSQYQEMQLQ**
801 **PLPSTSGPRA APPLPTQLQQ QQPPPPPPPP PPRQPGAAPA PLQFSYQTCE**
851 **LPSAASPAPD YPTPCQYPVD GAQQSDLTGP DCPR**SPGLQE APSSYDPLAL
901 SELPGLFDCE MLDAVDPQHN GYVLVN

#3

1 MVMADGPRHL QRGPVR**VGFY DIEGTLGKGN FAVVK**LGRHR ITKTEVAIK**I**
51 **IDKSQLDAVN LEK**IYR**EVQI MKMLDHPHII KLYQVMETKS MLYLVTEYAK**
101 **NGEIFDYLAN HGRLNESEAR RKFWQILSAV DYCHGR**KIVH R**DLKAENLLL**
151 **DNNMNIKIAD FGFGNFFK**SG ELLATWCGSP PYAAPEVFEG QQYEGPQLDI
201 WSMGVVLYVL VCGALPFDGP TLPILRQRVL EGR**FRIPYFM SEDCEHLIR**R
251 **MLVLDPSKRL TIAQIK**EHK**W MLIEVPVQRP VLYPQEQENE PSIGEFNEQV**
301 **LRLMHSLGID QQKTIESLQN KSYNHFAAIY FLLVER**LKSH R**SSFPVEQR**L
351 DGR**QRRPSTI AEQTVAKAQT VGLPVTMHSP NMR**LLR**SALL PQASNVEAFS**
401 **FPASGCQAEA AFMEEECVDT PKVNGCLLDP VPPVLVRKGC QSLPSNMMET**
451 **SIDEGLETEG EAEEDPAHAF EAFQSTR**SGQ R**RHTLSEVTN QLVVMPGAGK**
501 **IFSMNDSPSL DSVDSEYDMG SVQRDLNFLE DNPSLKDIML ANQPSPRMTS**
551 **PFISLRPTNP AMQALSSQK**R EVHNR**SPVSF R**EGR**RASDTS LTQGIVAFRQ**
601 **HLQNLARTKG ILELNKVQLL YEQIGPEADP NLAPAAPQLQ DLASSCPQEE**
651 **VSQQQESVST LPASVHPQLS PRQSLETQYL QHRLQKPSLL SKAQNTCQLY**
701 **CKEPPRSLEQ QLQEHR**LQQK RLFLQK**QSQL QAYFNQMQIA ESSYPQPSQQ**
751 **LPLPRQETPP PSQQAPPFSL TQPLSPVLEP SSEQMQYSPF LSQYQEMQLQ**
801 **PLPSTSGPRA APPLPTQLQQ QQPPPPPPPP PPRQPGAAPA PLQFSYQTCE**
851 **LPSAASPAPD YPTPCQYPVD GAQQSDLTGP DCPR**SPGLQE APSSYDPLAL
901 SELPGLFDCE MLDAVDPQHN GYVLVN

Supplementary Figure 3: Sequence coverage of HA-SIK2 in phosphopeptide mapping by LC-MS-MS. The sequence that was covered in Orbitrap LC-MS-MS analysis by peptides with Mascot scores indicating identity or extensive homology, ($p<0.05$; score >30 for (#1) and (#3) and >31 for (#2) is highlighted in bold and red (searches performed against SwissProt/Human database). Sequence coverage of HA-SIK2 was 79% from both unstimulated cells (#1), and glucagon-treated cells (#2) and 80% from insulin- treated cells (#3).

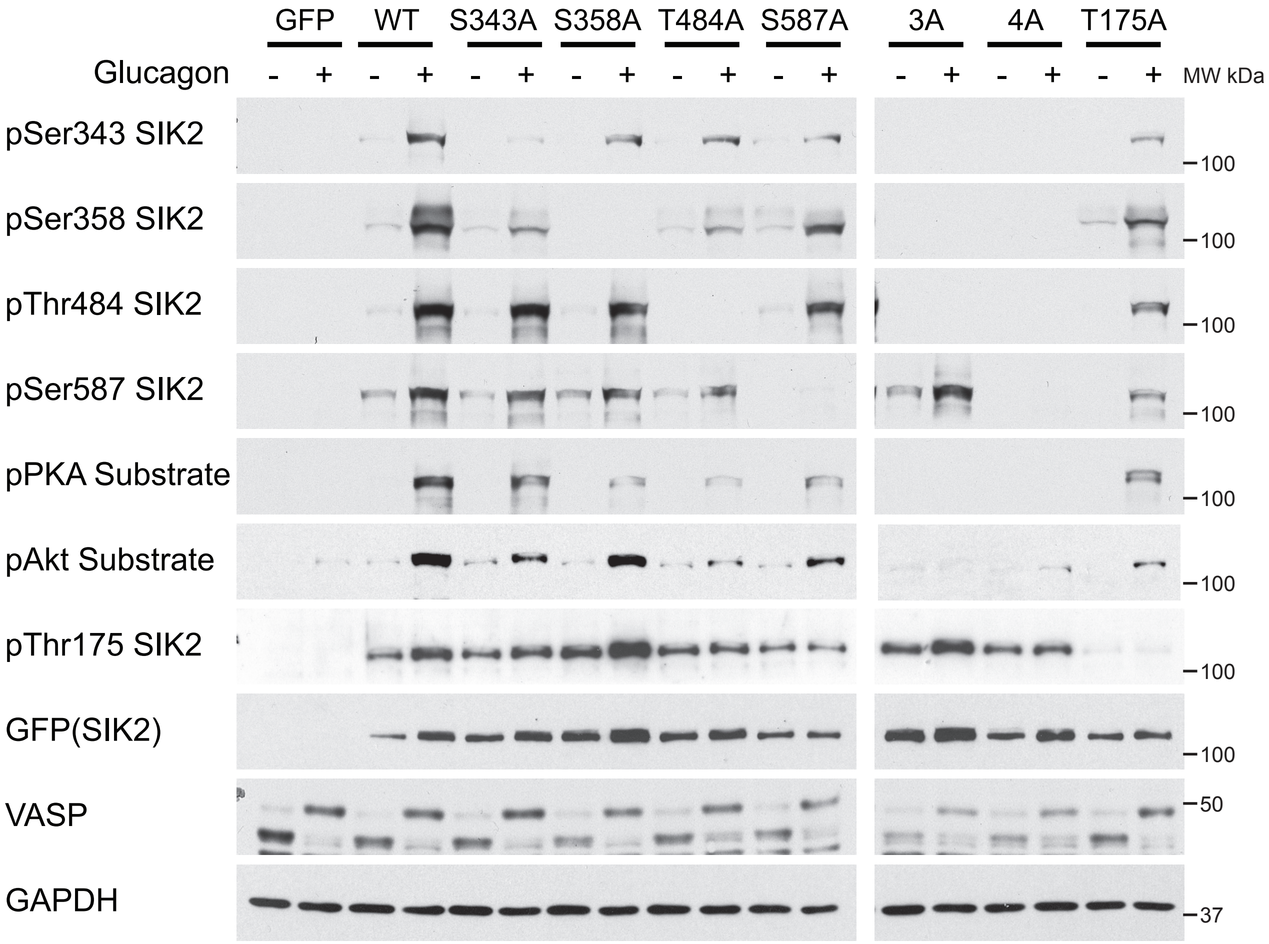

Supplementary Figure 4: Characterization of SIK2 phospho-specifi antibodies

Primary mouse hepatocytes (from 8-12 week-old male C57BL/6 mice) were infected with (1:2 MOI) adenoviral vectors encoding GFP-SIK2 wild-type or the indicated SIK2 phosphorylation-site mutants including 3A (S343A, S358A and T484A together), 4A mutant (3A and S587A together) and the kinase-inactive T-loop mutant T175A. Primary hepatocytes infected with GFP alone were included as a control. After 16 h the hepatocytes were stimulated for 10 min with 0.1 μM glucagon prior to cell lysis. The lysates were subjected to immunoblotting with the indicated phospho-specific SIK2 antibodies, with a phospho-PKA substrate motif antibody, with a VASP antibody to confirm the PKA activation by glucagon and with a GAPDH antibody as a loading control. Lysates were also immunoblotted with a phospho-Akt substrate motif antibody to compare the relative site selectivity with the phospho-PKA substrate motif antibody.

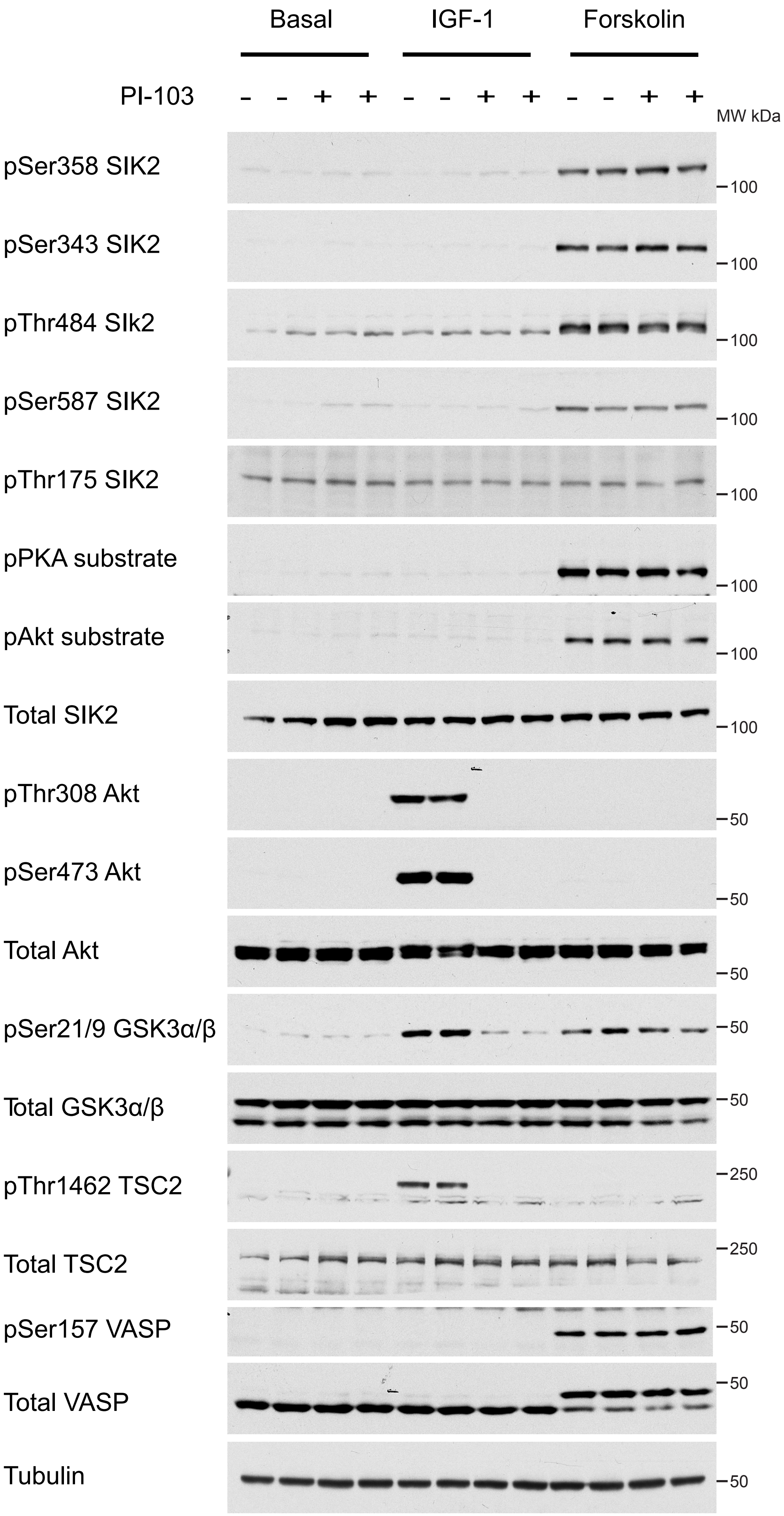

Supplementary Figure 5: Forskolin but not IGF-1 increases recombinant SIK2 phosphorylation
HA-SIK2 was ectopically expressed in HEK293 cells for 36 h followed by pre-treatment with the PI3K inhibitor PI-103 (1 µM for 30 min) and subsequently stimulated with 10 µM forskolin or 50 ng/ml IGF-1 prior to cell lysis. Lysates were immunoblotted with indicated antibodies. The pAkt, pGSK3α/β and pTSC2 antibodies were used to confirm the effects of IGF-1 and the inhibitor (PI-103) and VASP antibodies were used to confirm PKA activation by forskolin. The tubulin antibody was used as a loading control.

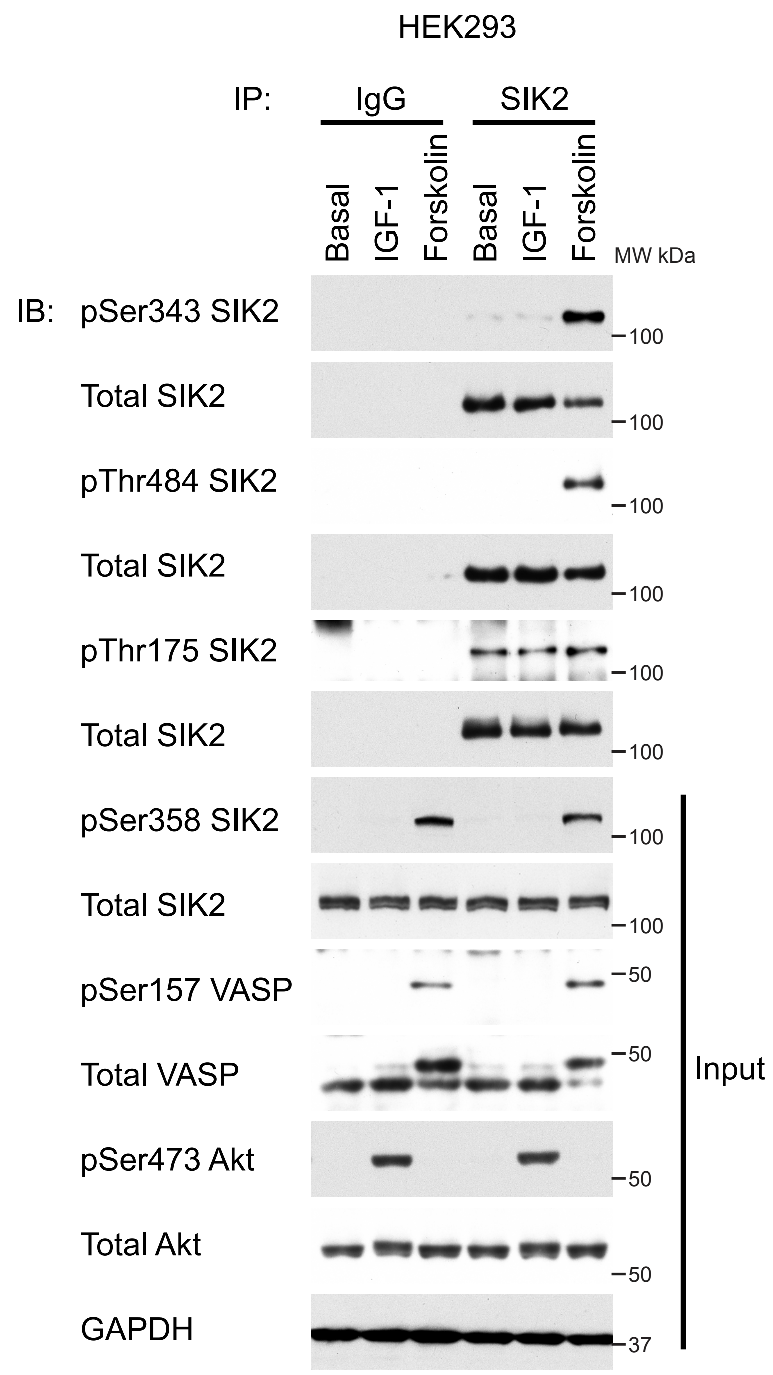

Supplementary Figure 6: Forskolin but not IGF-1 increases endogenous SIK2 phosphorylation
HEK293 cells were lysed following a 10-min treatment with 10 µM forskolin or 50 ng/ml IGF-1. Endogenous SIK2 was immunoprecipitated from 0.5 mg lysates (pre-immune IgG as a negative control). The precipitates were immunoblotted (along with 40 µg of lysates for input comparison) with the indicated SIK2 antibodies. The pAkt and VASP antibodies were used to confirm the stimulation by IGF-1 and forskolin respectively. The GAPDH antibody was used as a loading control.

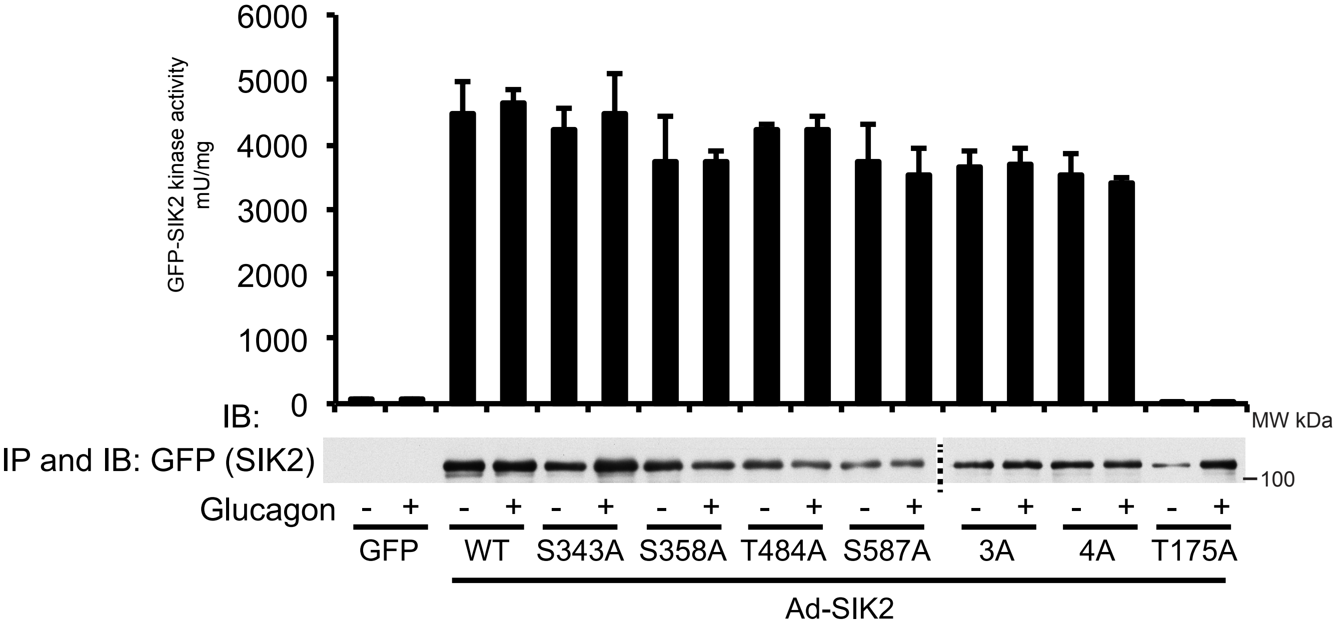

Supplementary Figure 7: Activity of SIK2 wild-type and phospho-defective mutants
GFP, GFP-SIK2 wild-type (WT) or the indicated GFP-SIK2 mutants were expressed in primary hepatocytes (from 8-12 week-old male C57BL/6 mice) using adenoviral vectors (1:2 MOI) for 16 h prior to 0.1 µM glucagon treatment for 10 min. GFP-SIK2 was immunoprecipitated from 50 µg of lysates with anti-GFP-agarose beads and subjected to either in vitro kinase assay or immunoblotting. The SIK2 3A recombinant protein included S343A, S358A and T484A mutations, while the 4A mutant had these mutations plus a S587A mutation. Data is presented as mean ± SD, n=3.

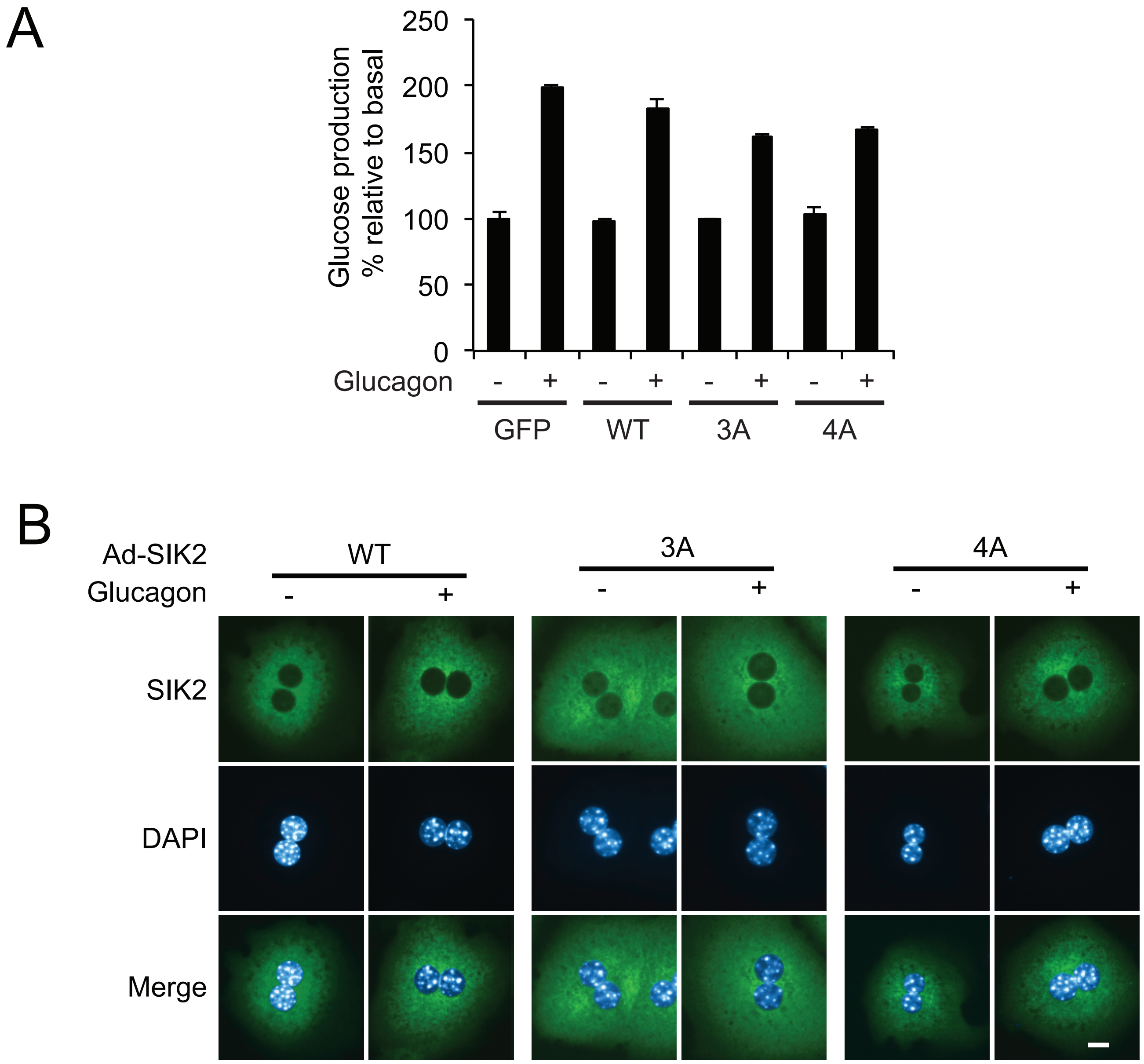

Supplementary Figure 8: Cellular localization of the SIK2 wild-type and the mutants
(A) Primary hepatocytes from 8-12 week-old male C57BL/6 mice were infected for total 20 h (1:1 MOI) with adenoviral vectors expressing GFP alone, GFP-SIK2 WT (WT), GFP- SIK2 3A (3A) or GFP-SIK2 4A (4A). The cells were treated for the last 8 h with 0.1 µM glucagon and the cell culture medium was used to measure glucose production. Glucose production was normalized to total protein content and presented relative to hepatocytes without treatment in GFP infected cells. Data is presented as mean ± SD, n=3. (B) Primary hepatocytes from 8-12 week-old male C57BL/6 mice were infected with the indicated SIK2 adenoviral constructs (Ad-GFP-SIK2) or GFP alone (MOI: 1:2) for 16 h followed by 0.1 µM glucagon stimulation for 10 min. Subsequently cells were fixed with 3.7% paraformaldehyde and immunostained with anti-SIK2 to visualize SIK2, or DAPI to visualize the nuclei. Images were captured with a fluorescence microscope (Nikon Eclipse Ti) using Nikon NIS-Elements BR 3.1 software. Data is representative of three independent experiments. Scale bar is 10 µm in length.

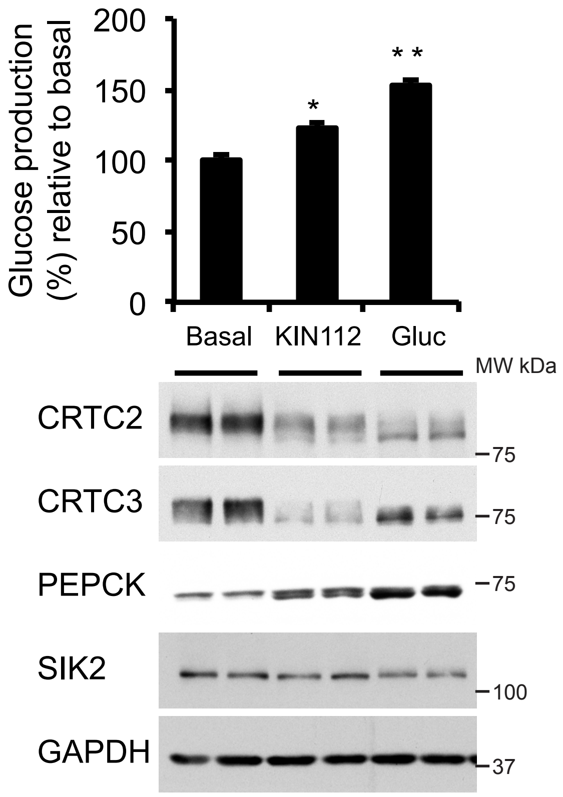

Supplementary Figure 9: Effect of KIN112 on phosphorylation of CRTCs and hepatic gluconeogenesis

Primary hepatocytes from 8-12 week-old male C57BL/6 mice were treated with 10 µM KIN112 (SIK inhibitor) or 0.1 µM glucagon (Gluc) for 12 h. Subsequently culture medium was used for measurement of glucose production (upper panel) while cells were lysed for immunoblotting (lower panels). Glucose production was normalized to total protein content and presented as a percentage of glucose production by hepatocytes without treatment (Basal). Lysates were immunoblotted with the indicated antibodies. Glucagon treatment was used as a positive control. Data is presented as mean ± SD, n=3. *$p<0.01$ basal versus KIN112, **$p<0.001$ basal versus glucagon.

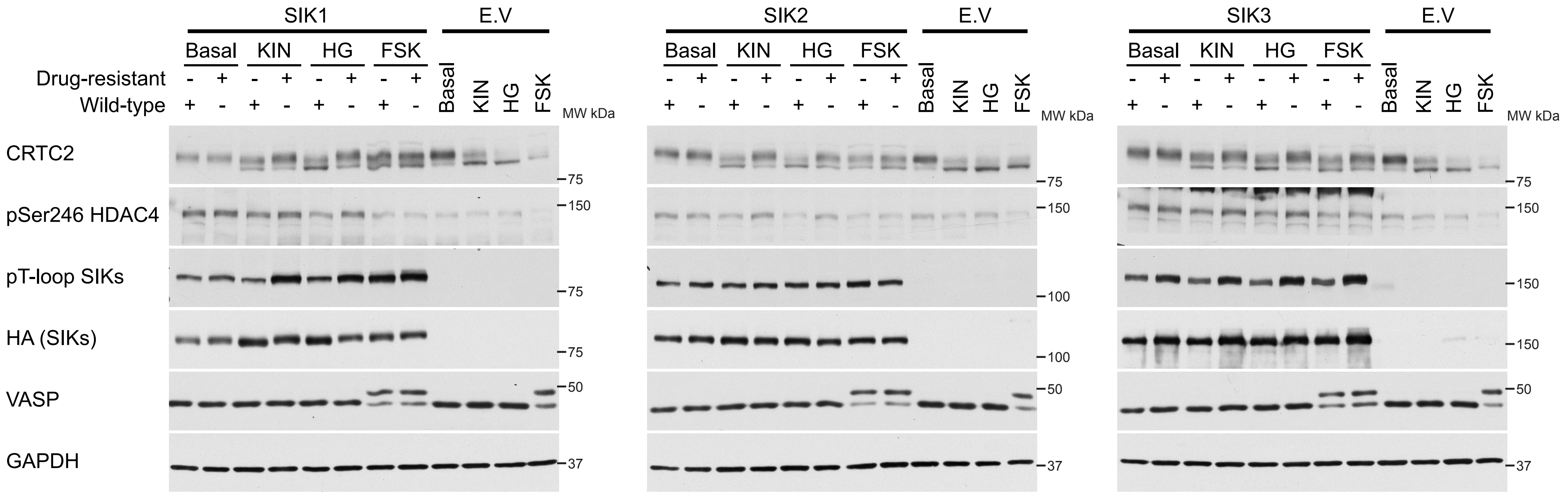

Supplementary Figure 10: SIK-isoform drug-resistant mutants in HEK293 cells
HA-empty-vector (E.V), HA-SIK wild-type or HA-SIK drug-resistant mutants (SIK1 T103Q, SIK2 T96Q and SIK3 T146Q) were expressed in HEK293 cells for 36 h followed by 1-h treatment with 10 μM KIN112 (KIN) or 2 μM HG-9-91-01 (HG) and 10 min stimulation with 10 μM forskolin (FSK) (as a positive control) prior to cell lysis. Lysates were immunoblotted with the indicated antibodies. The pT-loop SIK antibody recognizes the phosphorylation of an equivalent residue on SIK1 (T182), SIK2 (T175) and SIK3 (T221). The VASP antibody was used to confirm PKA activation by FSK and the GAPDH antibody was used as a loading control.

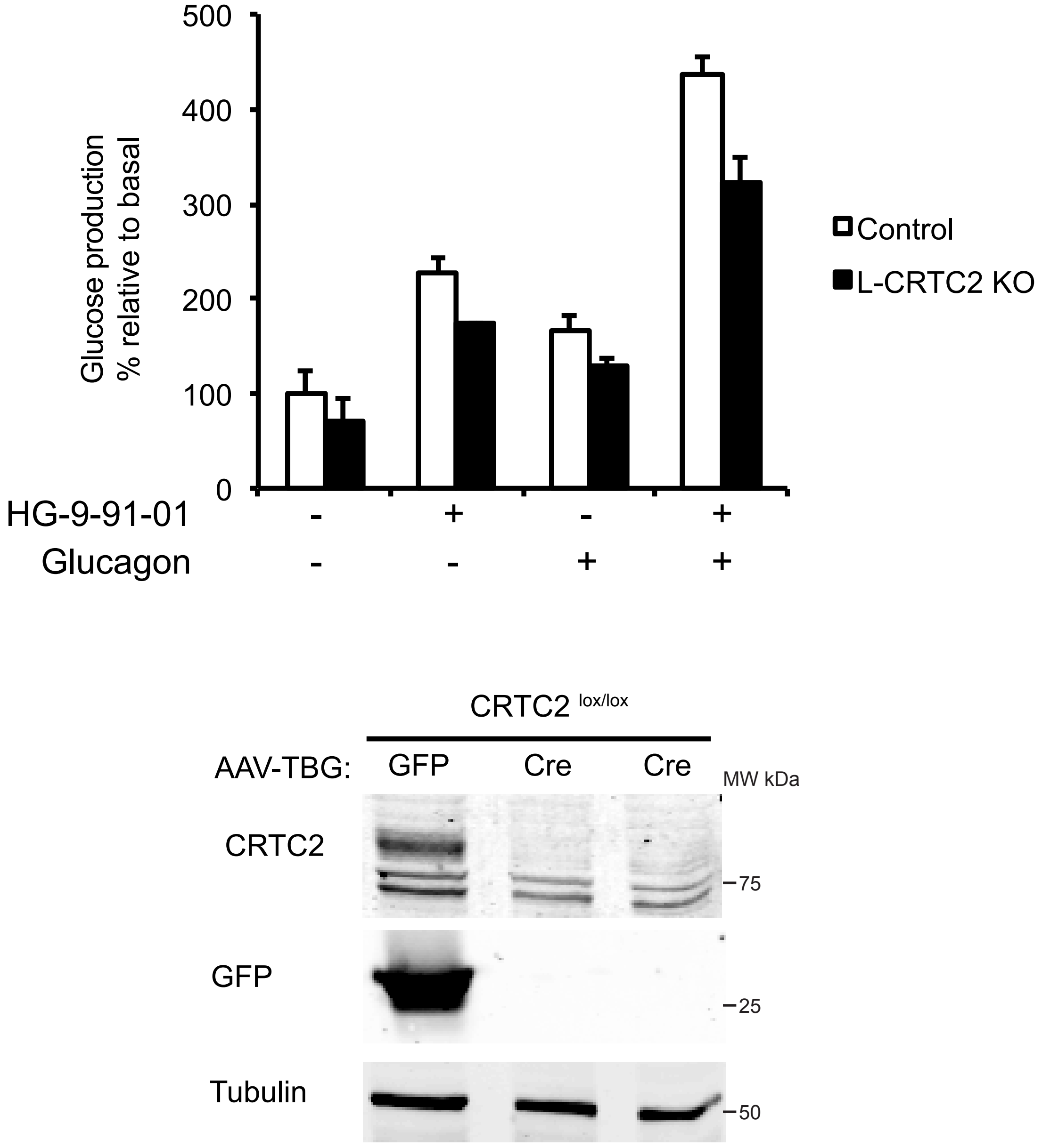

Supplementary Figure 11: Effect of the SIK inhibitor (HG-9-91-01) in CRTC2-deficient hepatocytes

(A) Glucose production from primary hepatocytes of control (CRTC2 lox/lox + AAV-TBG-GFP) and liver-specific (L)-CRTC2 KO (CRTC2 lox/lox + AAV-TBG-Cre) mice (8-12 week-old mice) 2 weeks following retro-orbital AAV injection was measured in response to 4 µM HG-9-91-01 or 0.1 µM glucagon treatment for 8 h. Glucose production was normalized to total protein content and presented as percentage of glucose production of control hepatocytes. (B) Lysates from the same experiment were immunoblotted with the indicated antibodies. Generation of CRTC2 lox/lox mouse has been described previously [1].

Supplementary Reference:

1. Le Lay J, Tuteja G, White P, Dhir R, Ahima R, Kaestner KH. CRTC2 (TORC2) contributes to the transcriptional response to fasting in the liver but is not required for the maintenance of glucose homeostasis. *Cell metabolism* **10**, 55-62 (2009).

Supplementary Figure 12: Original complete scans of the key immunoblots presented in the manuscript. Note that the data shown in manuscript is boxed. There are “cross outs” in some blots that are from the membranes stained with non-relevant antibodies exposed/scanned together with the membranes used for the manuscript.

Fig1C

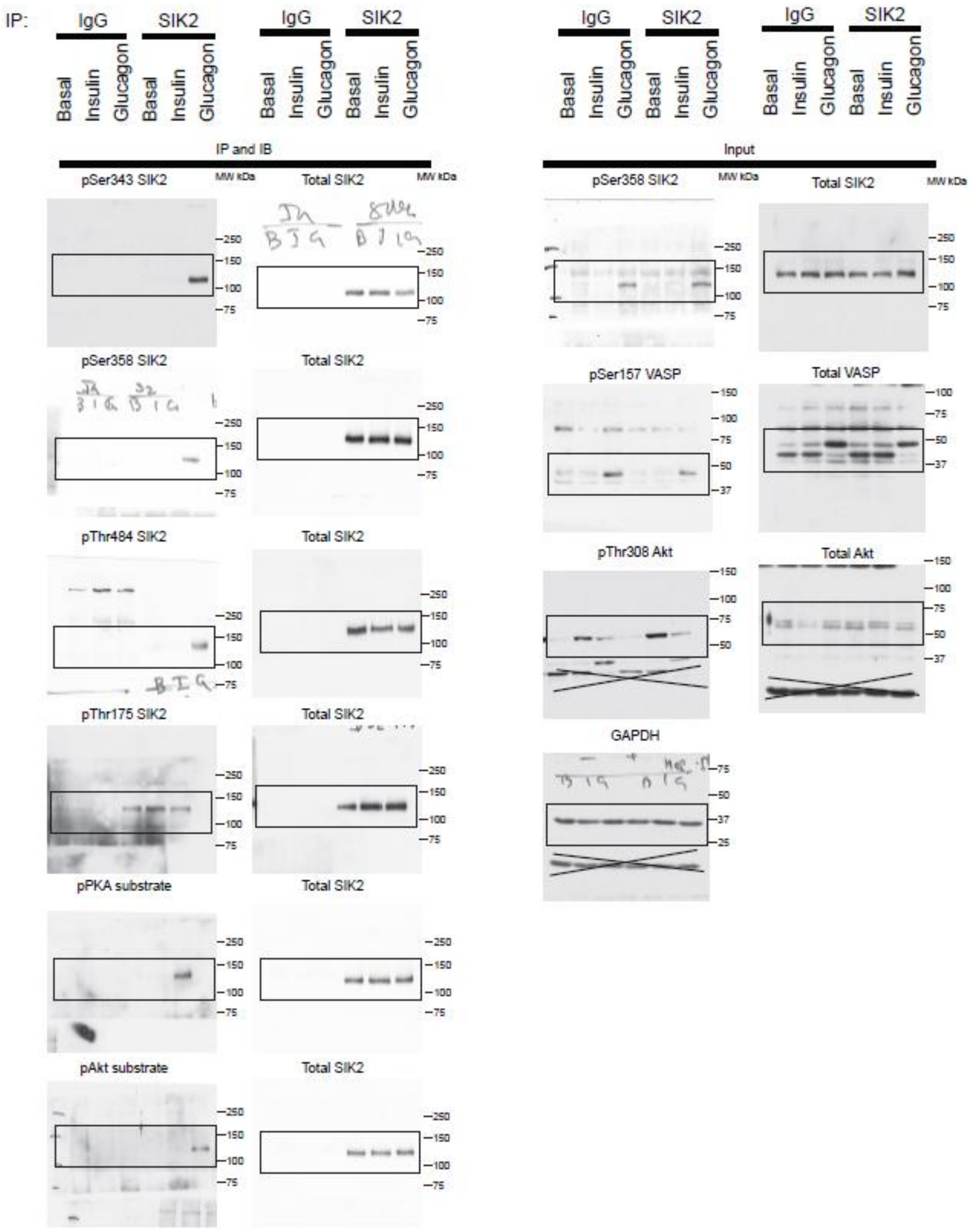

Fig2B

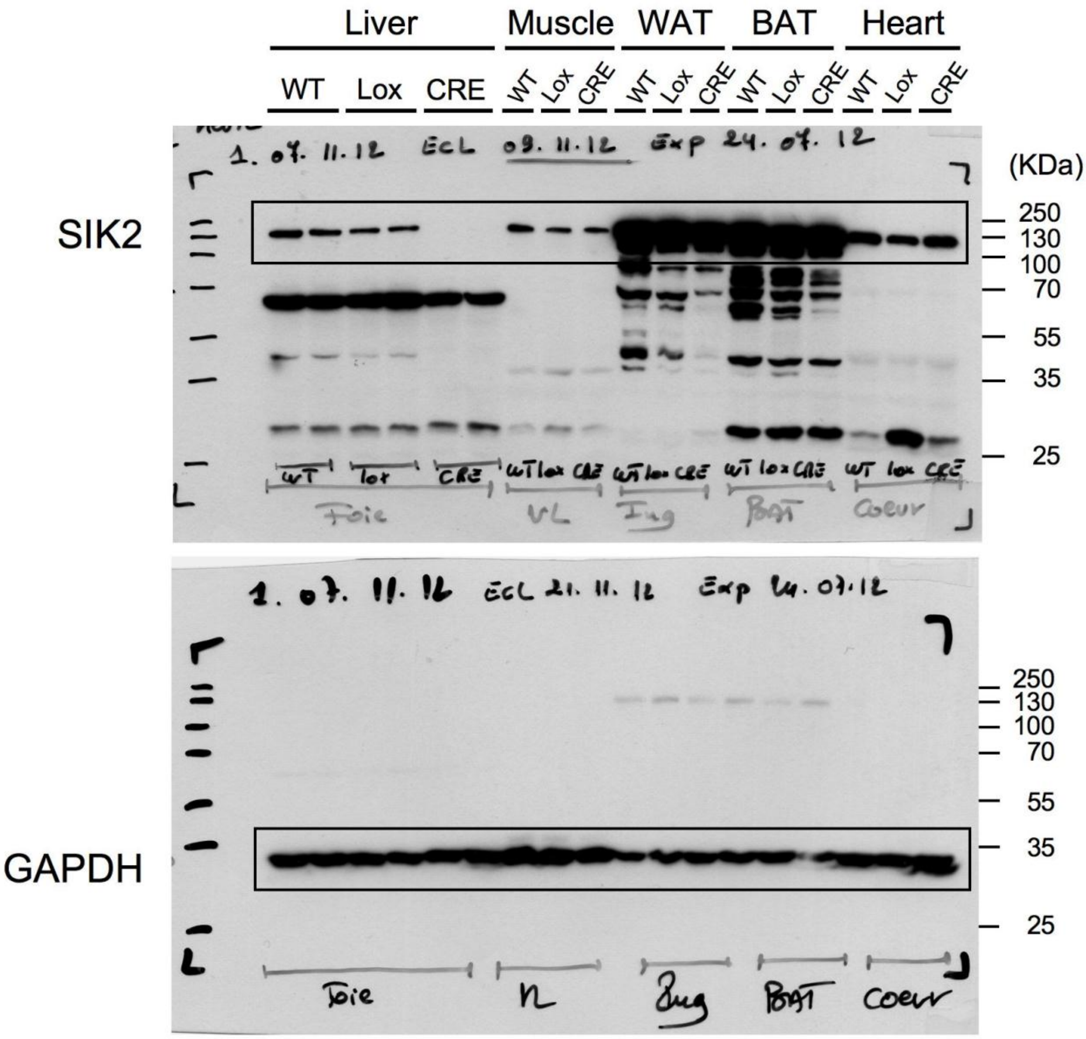

Liver
Muscle
WAT
BAT
Heart
WT
Lox
CRE
WT
Lox
CRE
WT
Lox
CRE
WT
Lox
CRE
WT
Lox
CRE
SIK2
(KDa)
250
130
100
70
55
35
25
GAPDH
250
130
100
70
55
35
25

Fig2G

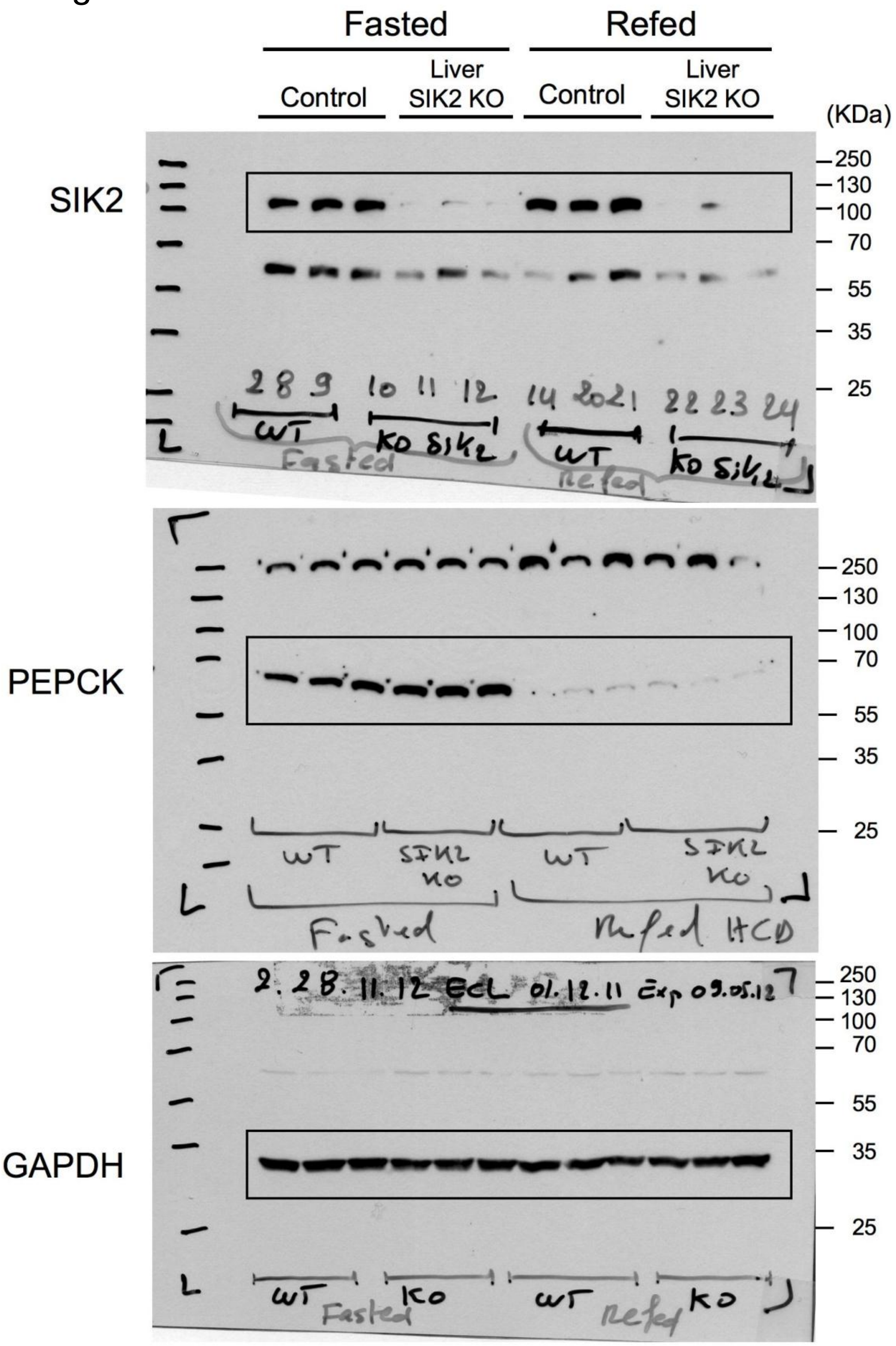
Fasted
Refed
Control
Liver SIK2 KO
Control
Liver SIK2 KO
(KDa)
SIK2
250
130
100
70
55
35
25
PEPCK
250
130
100
70
55
35
25
GAPDH
250
130
100
70
55
35
25

Fig3A

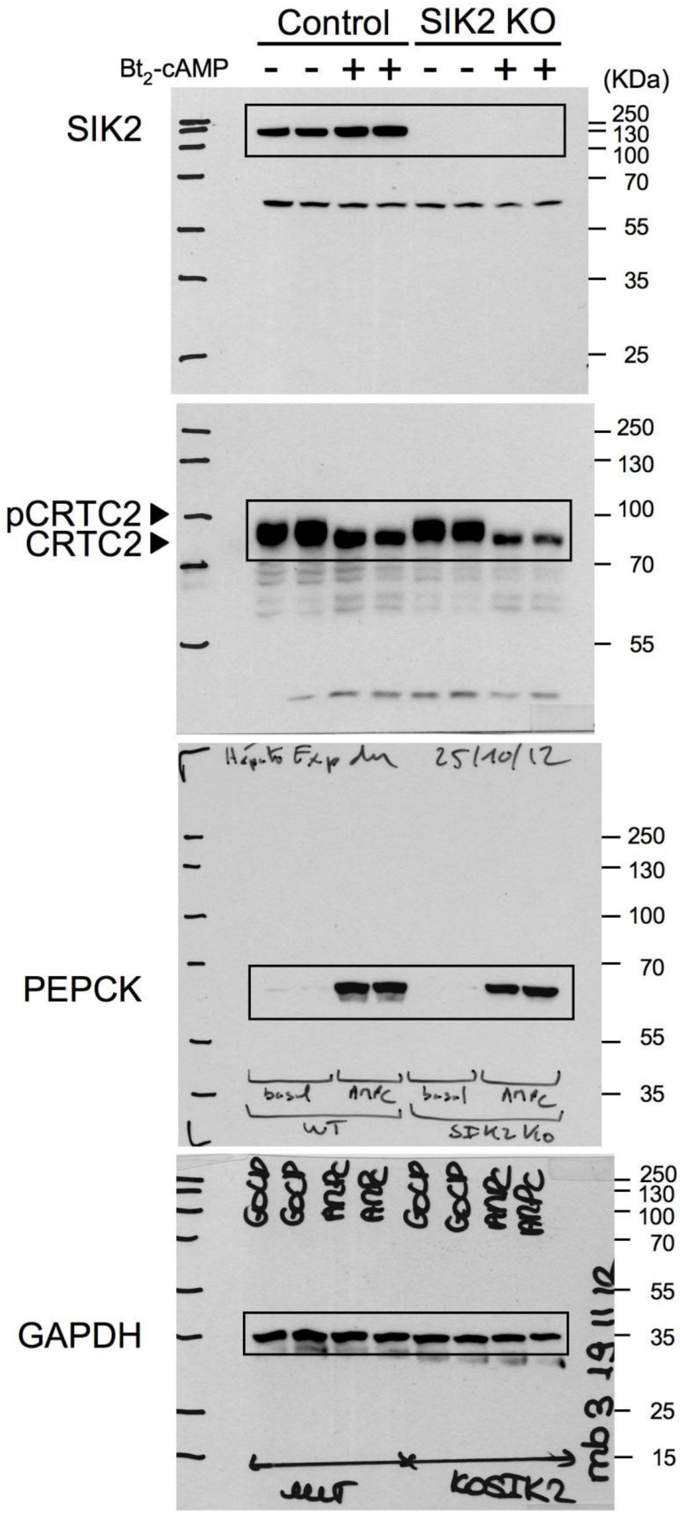
Control
SIK2 KO
Bt2-cAMP - - + + - - + +
(KDa)
SIK2
250
130
100
70
55
35
25
pCRTC2
CRTC2
250
130
100
70
55
PEPCK
250
130
100
70
55
35
GAPDH
250
130
100
70
55
35
25
15

Fig4B

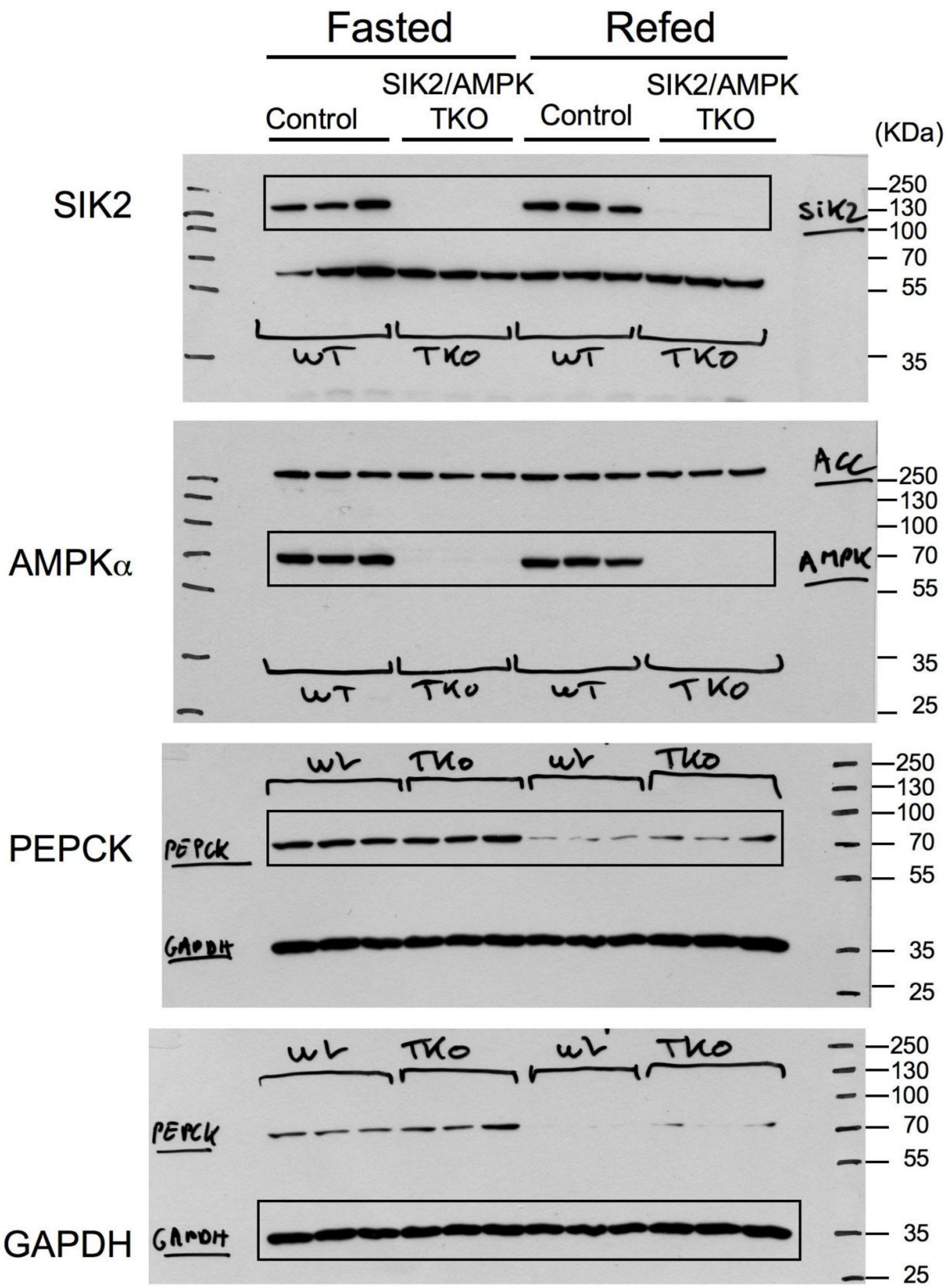
Fasted
Refed
Control
SIK2/AMPK TKO
Control
SIK2/AMPK TKO
(KDa)
SIK2
AMPKα
PEPCK
GAPDH
250
130
100
70
55
35
25

Fig5B

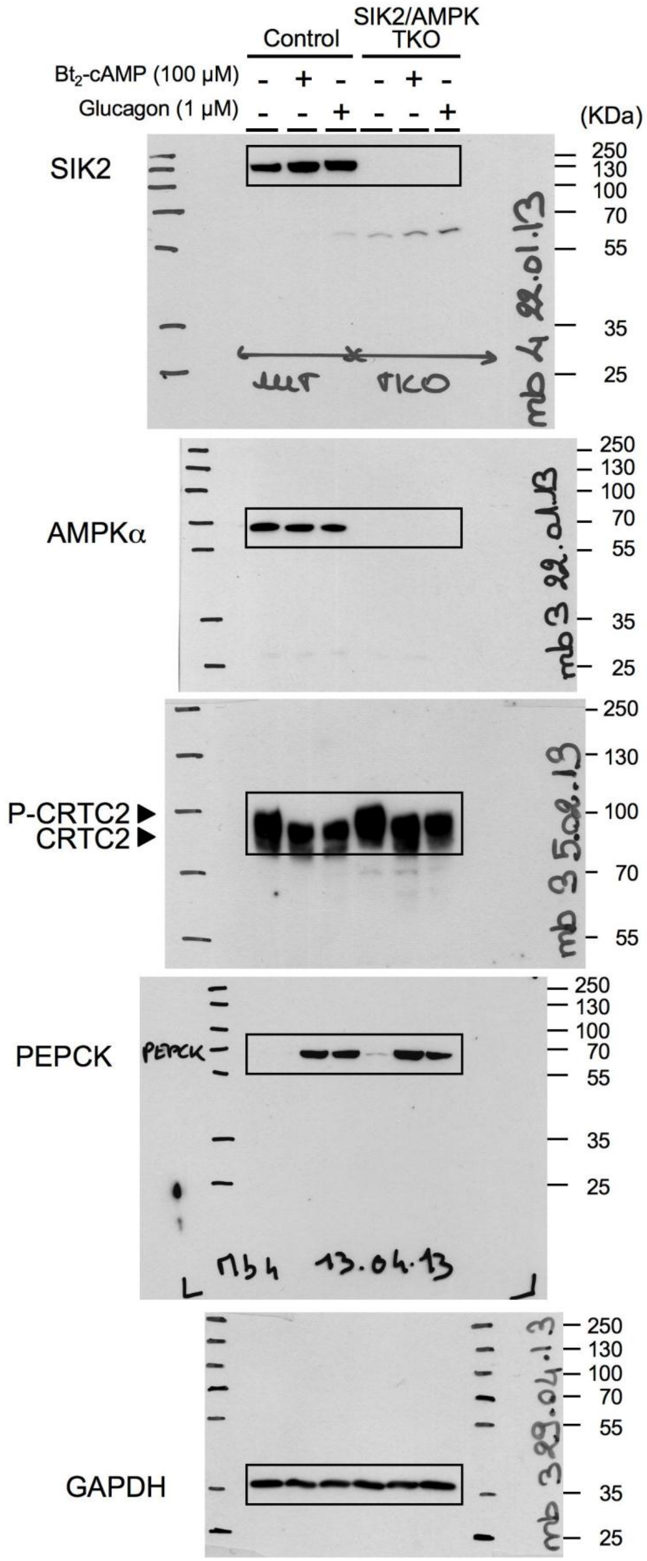
Control
SIK2/AMPK TKO
Bt2-cAMP (100 µM) - + - - + -
Glucagon (1 µM) - - + - - +
(KDa)
SIK2
AMPKα
P-CRTC2
CRTC2
PEPCK
GAPDH
250
130
100
70
55
35
25

Fig5C

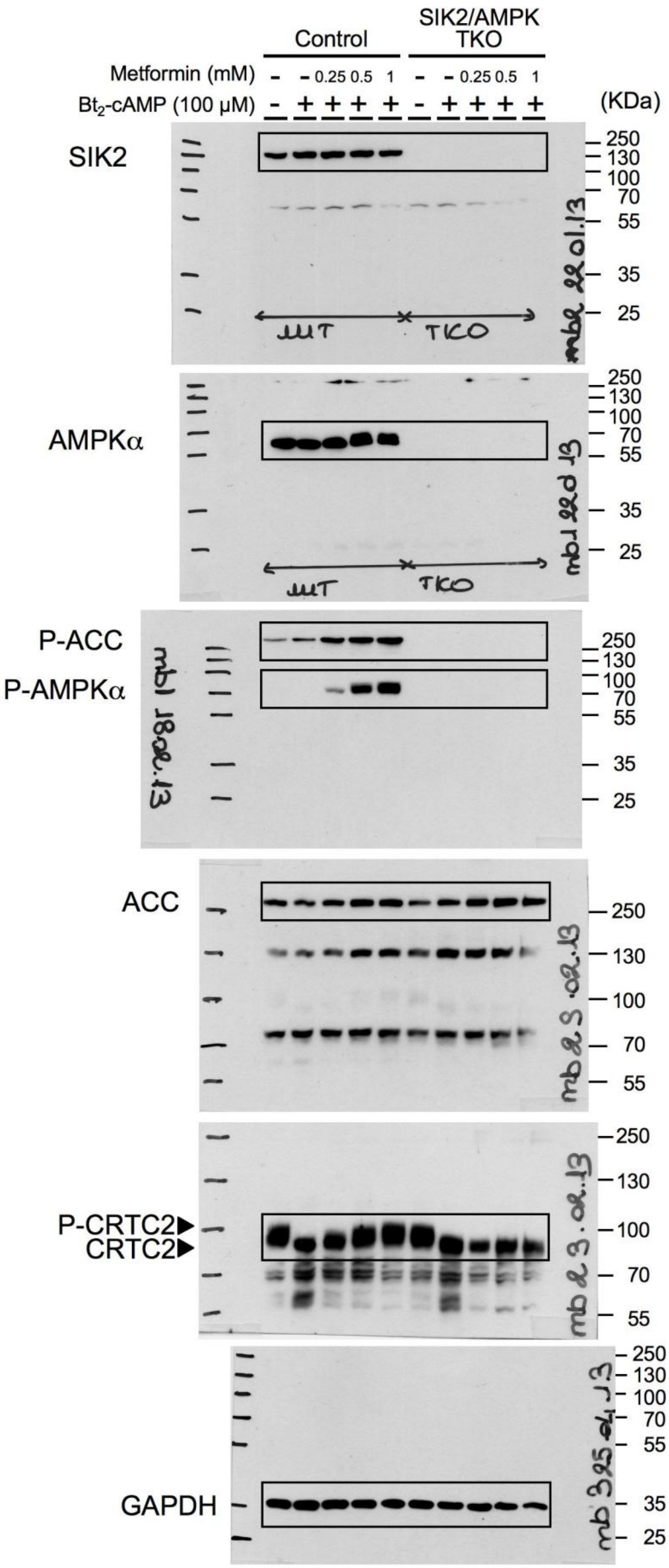
Control
SIK2/AMPK TKO
Metformin (mM) - - 0.25 0.5 1 - - 0.25 0.5 1
Bt2-cAMP (100 µM) - + + + + - + + + +
(KDa)
SIK2
250
130
100
70
55
35
25
AMPKα
250
130
100
70
55
35
25
P-ACC
P-AMPKα
250
130
100
70
55
35
25
ACC
250
130
100
70
55
P-CRTC2
CRTC2
250
130
100
70
55
GAPDH
250
130
100
70
55
35
25

Fig6A

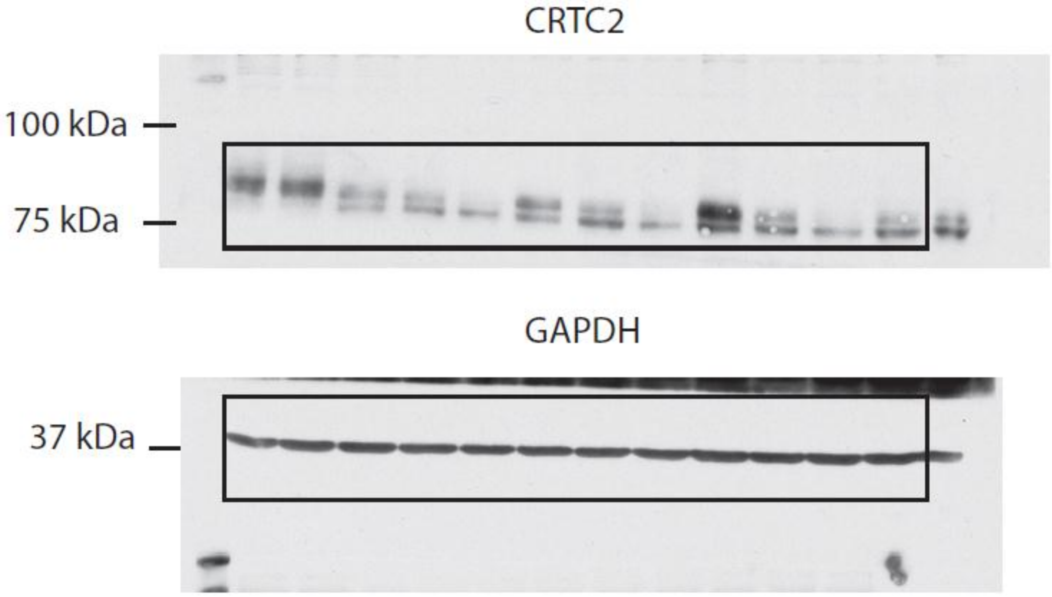
CRTC2
100 kDa
75 kDa
GAPDH
37 kDa

Fig6F

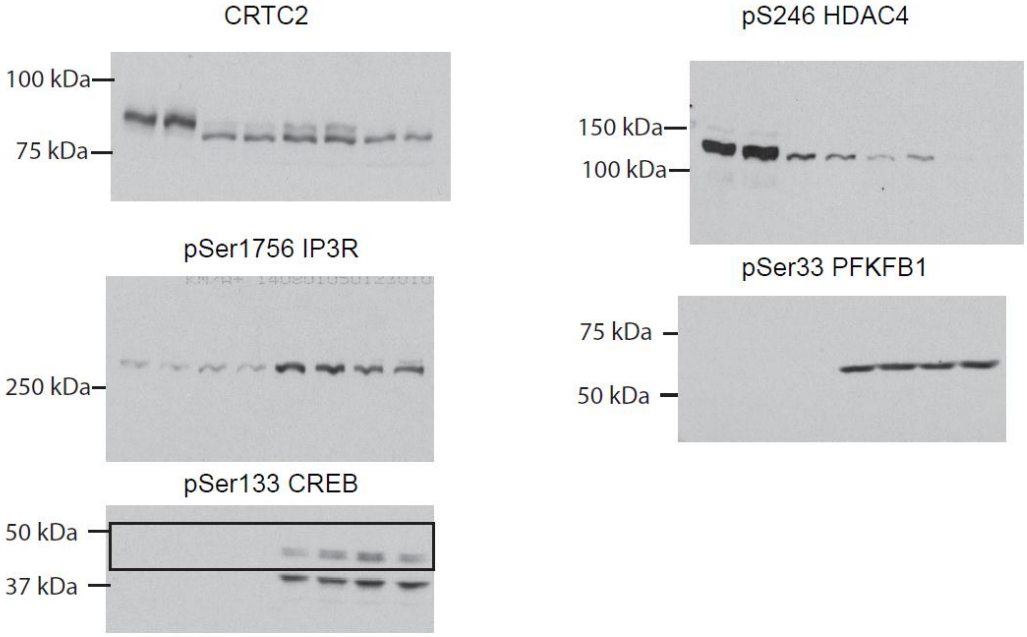
CRTC2
100 kDa
75 kDa
pS246 HDAC4
150 kDa
100 kDa
pSer1756 IP3R
250 kDa
pSer33 PFKFB1
75 kDa
50 kDa
pSer133 CREB
50 kDa
37 kDa

Fig7B

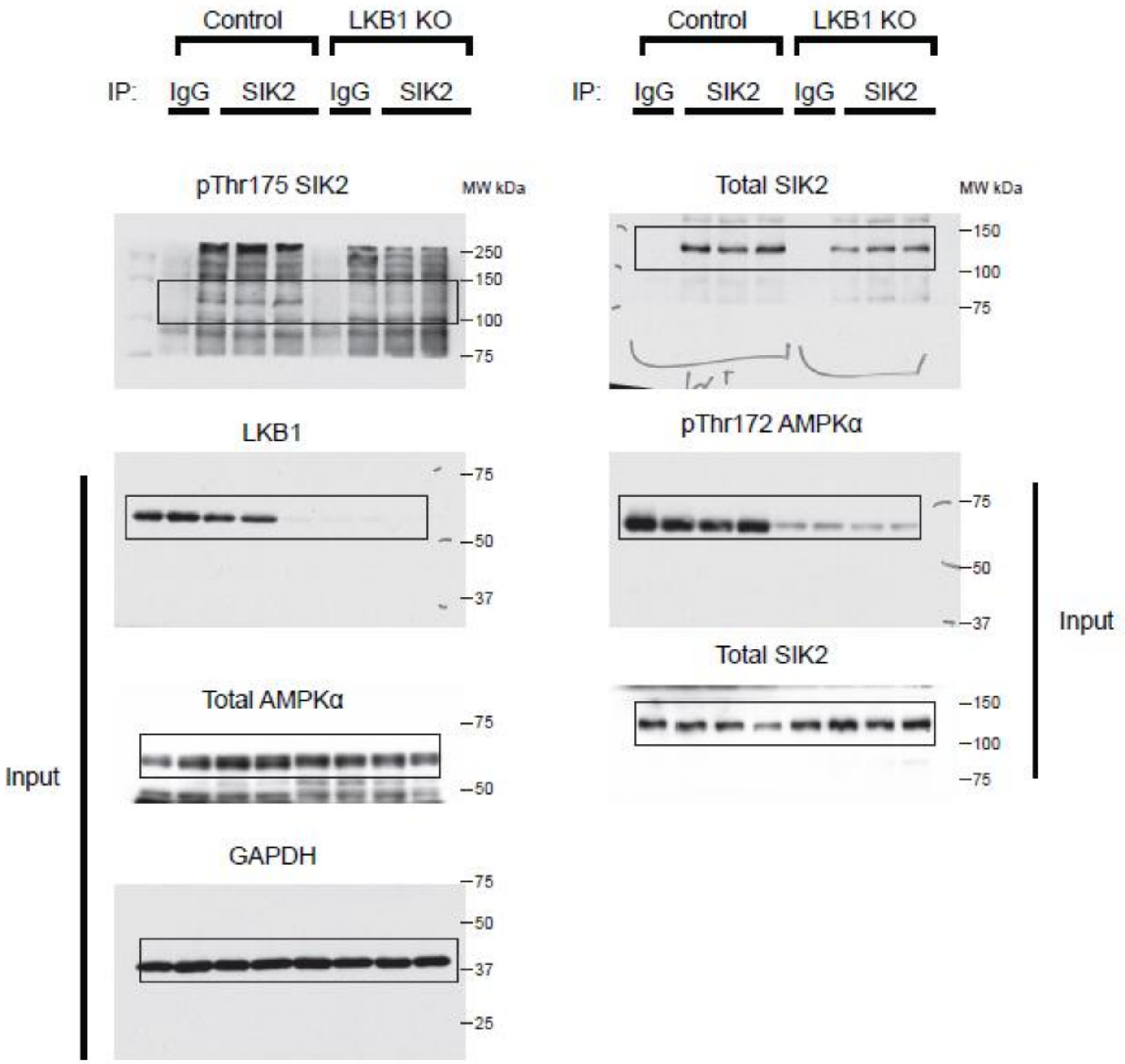
Control
LKB1 KO
IP:
IgG
SIK2
IgG
SIK2
pThr175 SIK2
MW kDa
250
150
100
75
LKB1
75
50
37
Total AMPKα
75
50
GAPDH
75
50
37
25
Input
Total SIK2
MW kDa
150
100
75
pThr172 AMPKα
75
50
37
Total SIK2
150
100
75
Input

Fig7C

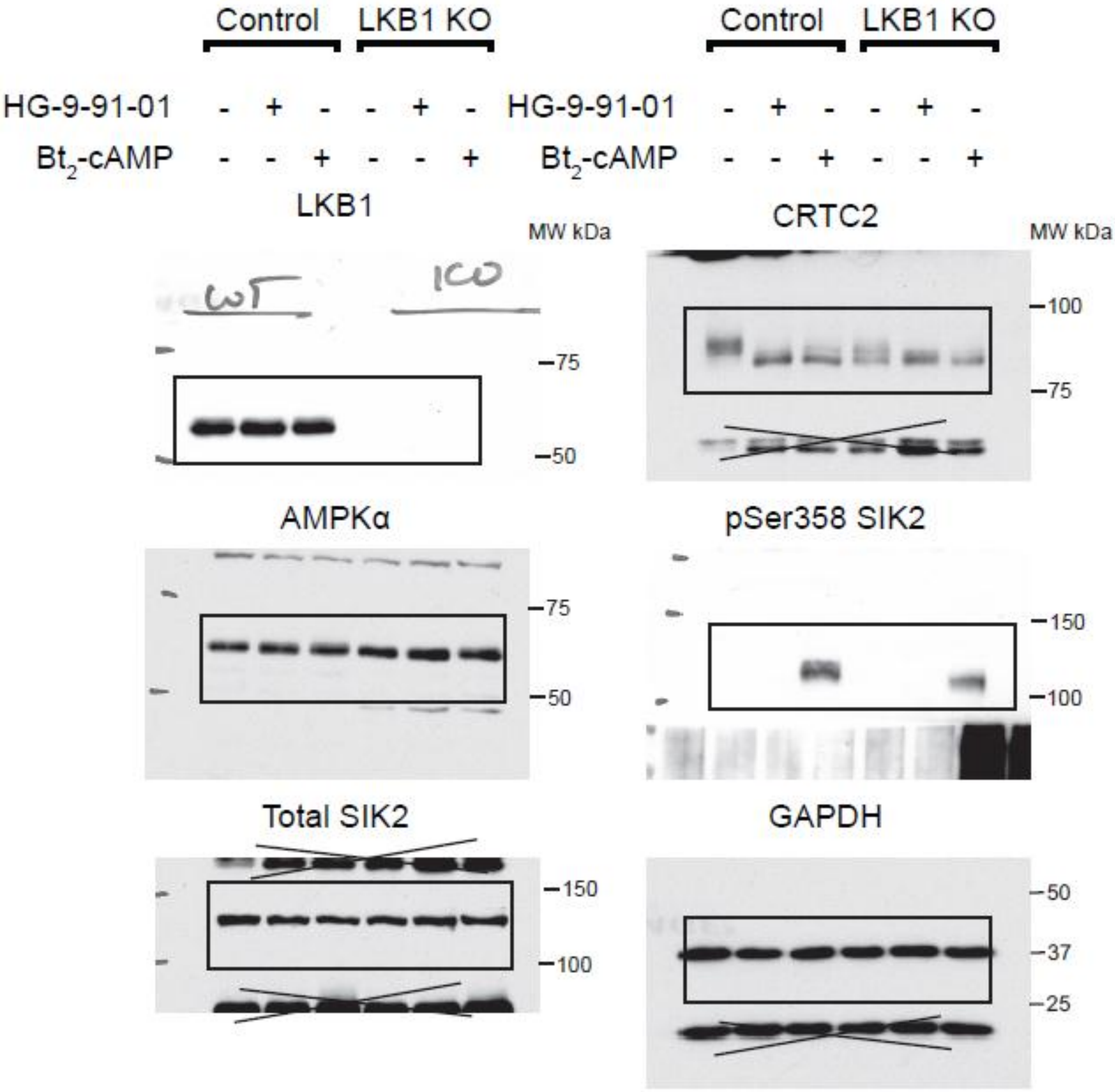
Control
LKB1 KO
HG-9-91-01 - + - - + -
Bt2-cAMP - - + - - +
LKB1
MW kDa
75
50
CRTC2
MW kDa
100
75
AMPKα
75
50
pSer358 SIK2
150
100
Total SIK2
150
100
GAPDH
50
37
25

Fig8B

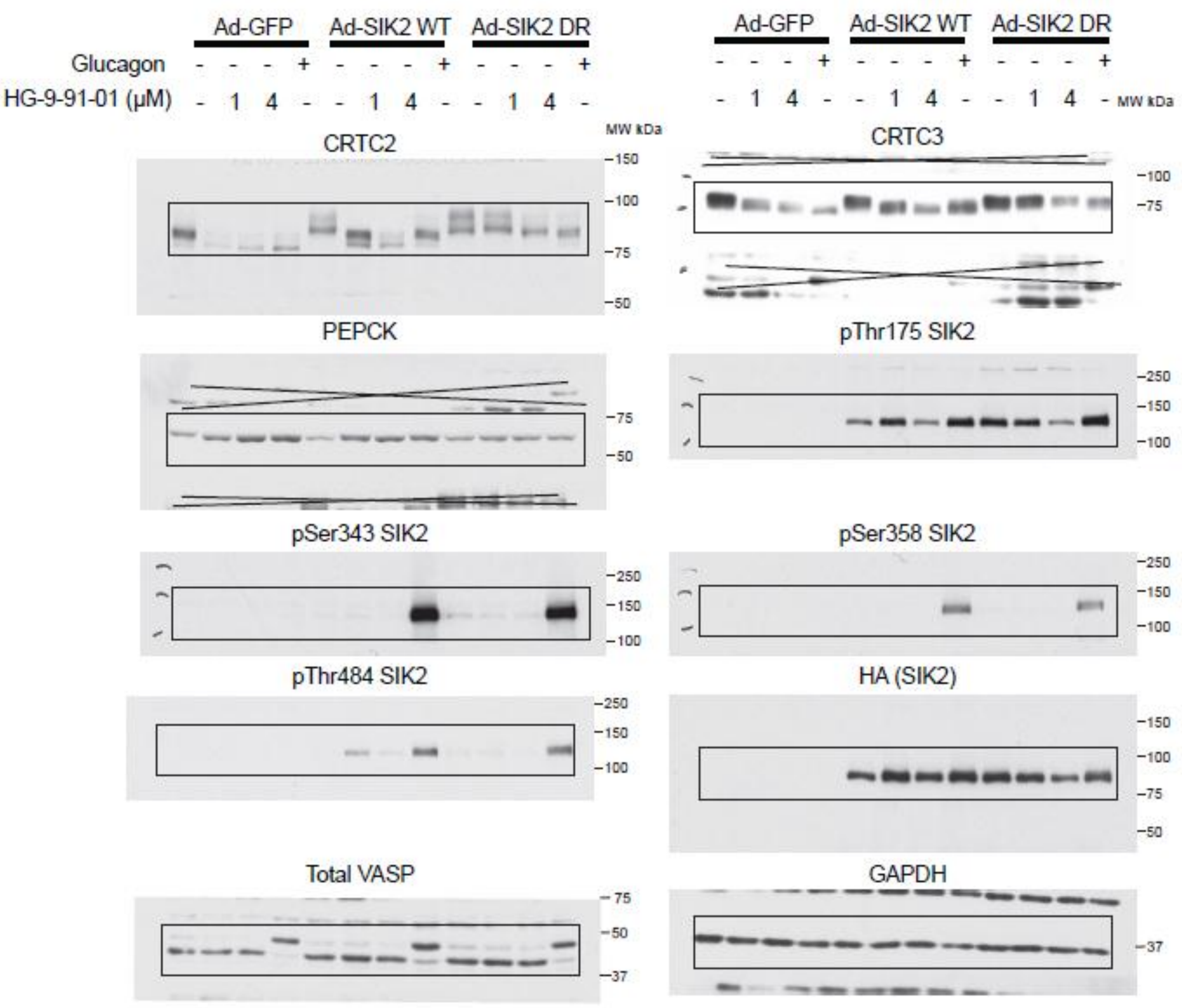
Ad-GFP
Ad-SIK2 WT
Ad-SIK2 DR
Glucagon
HG-9-91-01 (µM)
MW kDa
CRTC2
PEPCK
pSer343 SIK2
pThr484 SIK2
Total VASP
CRTC3
pThr175 SIK2
pSer358 SIK2
HA (SIK2)
GAPDH
